# Positive natural selection of N6-methyladenosine on the RNAs of processed pseudogenes

**DOI:** 10.1186/s13059-021-02402-2

**Published:** 2021-06-13

**Authors:** Liqiang Tan, Weisheng Cheng, Fang Liu, Dan Ohtan Wang, Linwei Wu, Nan Cao, Jinkai Wang

**Affiliations:** 1 Department of Medical Informatics, Zhongshan School of Medicine, Sun Yat-sen University, Guangzhou, 510080 China; 2 Center for Stem Cell Biology and Tissue Engineering, Key Laboratory for Stem Cells and Tissue Engineering, Ministry of Education, Sun Yat-sen University, Guangzhou, 510080 China; 3 Center for Biosystems Dynamics Research, RIKEN, 2-2-3 Minatojima-minamimachi, Chuo-ku, Kobe, Hyogo 650-0047 Japan; 4 Wuya College of Innovation, Shenyang Pharmaceutical University, Shenyang, 110016 China; 5 RNA Biomedical Institute, Sun Yat-sen Memorial Hospital, Sun Yat-sen University, Guangzhou, 510120 China

**Keywords:** Pseudogene, N6-methyladenosine, Natural selection, ceRNA, RNA stability

## Abstract

**Background:**

Canonical nonsense-mediated decay (NMD) is an important splicing-dependent process for mRNA surveillance in mammals. However, processed pseudogenes are not able to trigger NMD due to their lack of introns. It is largely unknown whether they have evolved other surveillance mechanisms.

**Results:**

Here, we find that the RNAs of pseudogenes, especially processed pseudogenes, have dramatically higher m^6^A levels than their cognate protein-coding genes, associated with de novo m^6^A peaks and motifs in human cells. Furthermore, pseudogenes have rapidly accumulated m^6^A motifs during evolution. The m^6^A sites of pseudogenes are evolutionarily younger than neutral sites and their m^6^A levels are increasing, supporting the idea that m^6^A on the RNAs of pseudogenes is under positive selection. We then find that the m^6^A RNA modification of processed, rather than unprocessed, pseudogenes promotes cytosolic RNA degradation and attenuates interference with the RNAs of their cognate protein-coding genes. We experimentally validate the m^6^A RNA modification of two processed pseudogenes, *DSTNP2* and *NAP1L4P1*, which promotes the RNA degradation of both pseudogenes and their cognate protein-coding genes *DSTN* and *NAP1L4*. In addition, the m^6^A of *DSTNP2* regulation of DSTN is partially dependent on the miRNA miR-362-5p.

**Conclusions:**

Our discovery reveals a novel evolutionary role of m^6^A RNA modification in cleaning up the unnecessary processed pseudogene transcripts to attenuate their interference with the regulatory network of protein-coding genes.

**Supplementary Information:**

The online version contains supplementary material available at 10.1186/s13059-021-02402-2.

## Background

Nonsense-mediated decay (NMD) is an important process for mRNA surveillance. It degrades mRNAs with premature translation termination codons (PTCs), which are usually generated *via* nonsense mutations, frameshift mutations, or aberrant splicing [1, 2]. NMD is critical for preventing the formation of truncated proteins, which could be poisonous to cells. Therefore, nonsense mutations that escape NMD often cause dominant-negative effects [1]. In yeast, PTCs are recognized by the presence of downstream sequence element (DSE), which can stimulate NMD [3]. However, the canonical NMD becomes a splicing-dependent process in mammals, which can be triggered as long as the stop codons are more than 50~55 bp upstream of the last exon-exon junction [1]. Although NMD can also be triggered by long 3′UTRs [4], intronless genes are likely insensitive to NMD [5, 6].

As non-functional copies of closely related protein-coding genes, pseudogenes are one of the major class of substrates of NMD due to their accumulated nonsense mutations in the evolution [7, 8]. Although in most cases pseudogenes may still not be very important, increasing evidence indicates that some pseudogenes play important regulatory roles on regulating their protein-coding cognates; dysregulation of pseudogenes are associated with various human diseases including cancer [9]. Due to the high sequence similarities with their protein-coding cognates, pseudogenes often work as competitive endogenous RNAs (ceRNAs), which competitively bind microRNAs (miRNAs) or RNA binding proteins (RBPs) to prevent the degradation (or other processes) of their cognate protein-coding genes, such as *PTENP1* [10], *BRAFP1* [11], and *HMGA1* [12]. Some other pseudogenes can also generate endogenous small interfering RNAs (esiRNA), such as *PPM1K* [13], or work as trans-acting antisense RNAs, such as *nNOSP* [14].

There are three classes of pseudogenes according to the unique biogenesis mechanisms: unitary pseudogenes, unprocessed pseudogenes, and processed pseudogenes [15]. Unitary pseudogenes are single-copy genes with spontaneous mutations in the coding regions or regulatory regions, resulting in genes unable to be transcribed or translated into proteins. Unprocessed pseudogenes are originated through gene duplications and subsequent mutations that cause frameshifts or early terminators. Processed pseudogenes, also known as retrotransposed pseudogenes, are generated through retrotransposition of mRNA transcripts, thus do not have introns but may have poly(A) tails. As previously reported, the retrotransposed mRNAs tend to be stable transcripts translated on free cytoplasmic ribosomes [16]. Because canonical NMD is a splicing-dependent process in mammals, processed pseudogenes are not likely the substrates of NMD due to the lack of introns. It is largely unknown whether processed pseudogenes are subject to other RNA surveillance pathways and whether they have acquired novel surveillance mechanisms in the evolutionary history since they diverged from their cognate protein-coding genes.

N6-methyladenosine (m^6^A) RNA modification is reported in recent years as a novel pathway of degrading RNAs [17, 18]. m^6^A is a reversible and prevalent internal RNA modification in mRNAs and long noncoding RNAs (lncRNAs). It is installed on “DRACH” motifs of RNAs [19] by m^6^A methyltransferases complex with METTL3 as the catalytic subunit [17, 18]. Demethylases FTO and ALKBH5 can reverse the modification [17, 18]. In addition, m^6^A can be specifically regulated through a variety of RNA binding proteins and co-transcriptionally through transcription factors as well as H3K36me3 histone modification [17, 18, 20]. Upon m^6^A modification on mRNAs, m^6^A readers such as YTH domain-containing proteins can specifically read the m^6^A and regulate various post-transcriptional processes of host mRNAs [17, 18], such as promoting the cytosolic degradation [21–23] and nuclear export of mRNAs [24]. In recent years, critical roles of m^6^A have been reported in a variety of physiological and pathological processes [25–27].

Based on the genome-wide profiling of m^6^A, the RNAs of pseudogenes are also modified by m^6^A [28]. However, little is known about the function of the m^6^A sites on pseudogenes and how they have evolved after separating from their cognate protein-coding genes.

In this study, we found the RNAs of human processed pseudogenes were accumulating novel m^6^A modifications in company with novel m^6^A motifs after separating with their cognate protein-coding genes. We found convergent evidence supporting that these recently accumulated m^6^A motifs had evolved under positive selection. Based on bioinformatic analyses and experimental validation, we have revealed that these m^6^A sites on the RNAs of processed pseudogenes promoted cytosolic RNA degradation and attenuated their unnecessary interfering with their cognate mRNAs. Our discovery illustrates the evolutionary landscape of an m^6^A-mediated RNA surveillance mechanism for NMD resistant RNAs.

## Results

### The RNAs of pseudogenes tend to have higher m^6^A levels than their cognate mRNAs

In order to study the functions and evolution of m^6^A on the RNAs of pseudogenes, we first ask whether the RNAs of pseudogenes are methylated differently from their cognate mRNAs. We previously developed m^6^A-LAIC-seq technology to quantify the m^6^A levels in transcriptome-wide scale [28]. Here, we took advantage of our previously published m^6^A-LAIC-seq data of GM12878, which is a human B-lymphoblastoid cell line sequenced deeply in 1000 genome project, as well as H1, which is a human embryonic stem cell line, to study the m^6^A levels of the RNAs of pseudogenes [28].

Since pseudogenes and their cognate protein-coding genes have similar sequences, cross-mapping can happen frequently when mapping the short-reads of next-generation sequencing to the genome, which will distort the expression patterns of pseudogenes. Here, we improved the mapping procedure to minimize the occurrence of cross-mapping by only allowing perfect matches or mismatches at known SNPs when we aligned the reads to hg19 human genome using HISAT2 [29]. Because the SNPs of GM12878 were mostly included in the known SNP database, the new procedure is more powerful for GM12878 and we mainly focused on this cell line for the downstream analyses. Compared with the conventional HISAT2 mapping procedure allowing 3 mismatches, the new procedure resulted in a reduced number of mapped reads for a large number of genes, reflecting the stricter mapping criteria of the new mapping procedure. In contrast, we observed similar numbers of pseudogenes with more or less mapped reads using the new mapping procedure, suggesting that the stricter new procedure cause re-assignment of mapped reads on pseudogenes and their homologous genomic loci compared with the conventional procedure (Additional file 1: Figure S1a-c). As shown in Additional file 1: Figure S1d and e, we observed dramatic read coverage changes in certain regions of pseudogene *RPS7P1* based between the two mapping procedures, due to the re-assignment of reads from its protein-coding cognate *RPS7* to pseudogene *PRS7P1* in the new mapping procedure; in another case, some reads were re-assigned from pseudogene *PPP1R14BP3* to its cognate protein-coding gene *PPP1R14B* in the new procedure.

**Fig. 1:**
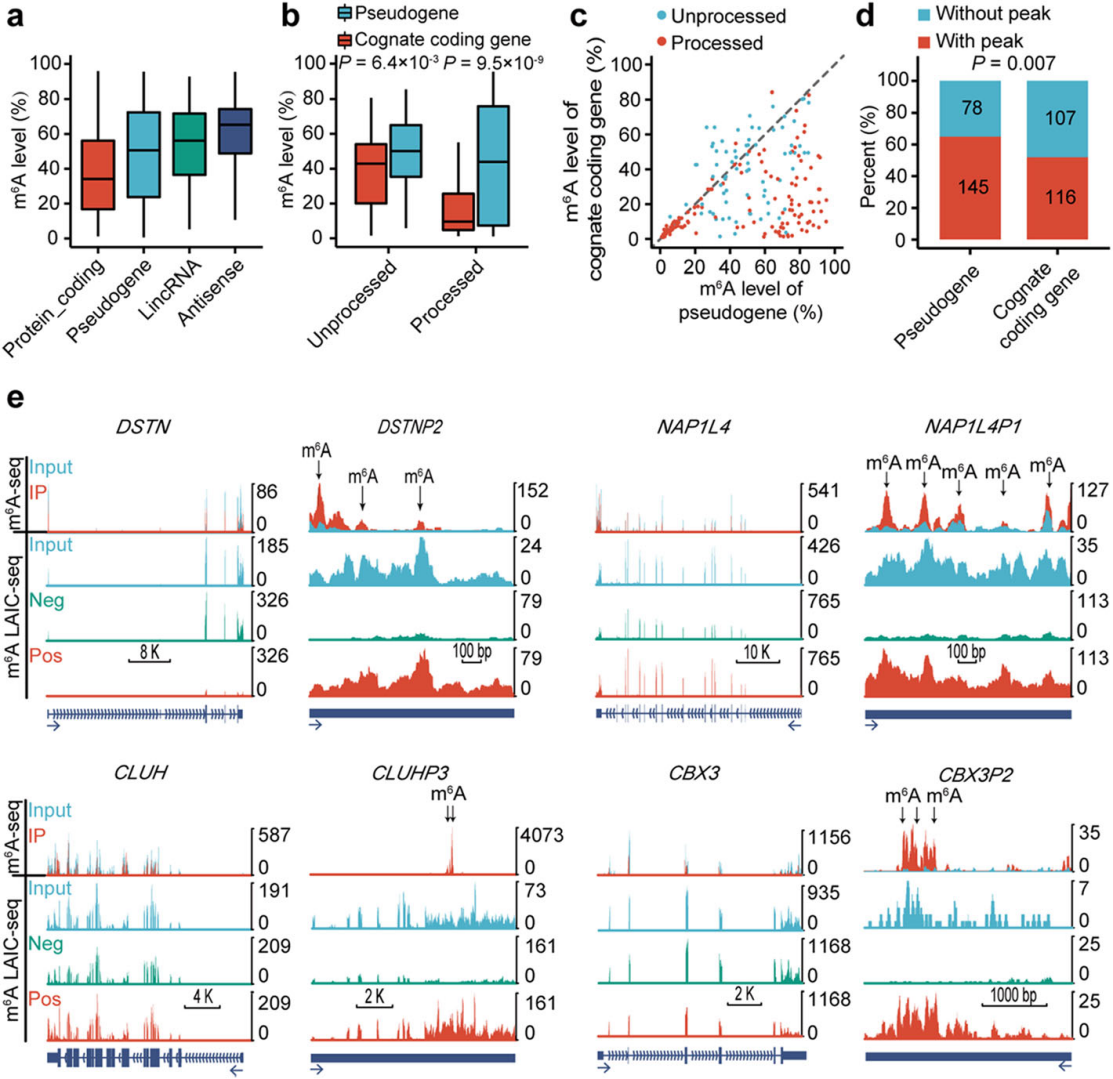
Comparisons of m^6^A between pseudogenes and their cognate protein-coding genes in GM12878 cell line. **a** Box plot comparing the m^6^A levels among different biotypes. **b** Box plot comparing the m^6^A levels between pseudogenes and their cognate protein-coding genes for processed pseudogenes and unprocessed pseudogenes respectively. **c** Scatter plot comparing the m^6^A levels between pseudogenes and their cognate protein-coding genes for processed pseudogenes and unprocessed pseudogenes respectively. **d** Stacked bar plot comparing the percent of pseudogenes and their cognate protein-coding genes with or without m^6^A peaks. The frequencies were displayed in the corresponding boxes. **e** UCSC genome browser tracks of m^6^A-seq and m^6^A-LAIC-seq data showing representative examples of pseudogenes and their cognate protein-coding genes. Read-coverage tracks of input, m^6^A-negative, and m^6^A-positive fractions of m^6^A-LAIC-seq shown along with overlay tracks of m^6^A-seq (red for RIP and cyan for input; predicted m^6^A sites in m^6^A peaks are indicated by arrows). Read coverage (y-axis) of m^6^A negative and m^6^A positive are normalized as previously described [28] to reflects the calculated m^6^A levels (i.e., equal signals in m^6^A positive (eluate) versus m^6^A negative (supernatant) indicate m^6^A levels of 50%), while input and m^6^A-seq tracks are shown for optimal viewing

We took advantage of the annotations from GENCODE, Vega, and psiCube [30] databases to compile a list of 12,143 one-to-one pairs of pseudogenes and corresponding protein-coding genes, including 4078 protein-coding genes with different numbers of pseudogenes (Additional file 2: Table S1). Among them, only 223 transcribed pseudogenes with reliable m^6^A levels were detected in the m^6^A-LAIC-seq data of GM12878 cell line, reflecting that most pseudogenes are not transcribed [31]. The cognate protein-coding genes of these pseudogenes were enriched in mRNA catabolic process, endoplasmic reticulum localization, and translational initiation, consistent with the previous report about transcribed pseudogene [9] (Additional file 1: Figure S2a, b). We found m^6^A levels of pseudogenes were higher than protein-coding genes, but lower than lincRNA and antisense RNAs (Fig. 1a).

We then performed a pairwise comparison between the m^6^A levels of pseudogenes and their cognate protein-coding genes. We found the m^6^A levels of pseudogenes were much higher than their cognate protein-coding genes, the differences were much more dramatic in processed pseudogenes than unprocessed pseudogenes (Fig. 1b, c). Consistently, it was more dramatic in intronless pseudogenes than pseudogenes with multiple exons (Additional file 1: Figure S2c, d; Additional file 2: Table S2). Similar results were observed in H1 hESC based on 190 pseudogenes with reliable m^6^A levels (Additional file 1: Figures S2e, f and S3a-c; Additional file 2: Table S3). These results suggest that the m^6^A of pseudogenes may get promoted in the evolution after separation from their cognate protein-coding genes.

Because m^6^A-LAIC-seq quantifies the m^6^A at gene level, we used the m^6^A-seq data of GM12878 [32] to test whether the elevation of m^6^A levels on pseudogenes was due to de novo formation of m^6^A peaks on pseudogenes or facilitated methylation on ancestral m^6^A sites. We found 65% of the pseudogenes having m^6^A peaks; in contrast, 52% of their cognate protein-coding genes had m^6^A peaks (*P* = 0.007, two-tailed chi-square test) (Fig. 1d). As shown in Fig. 1e, protein-coding gene *DSTN* does not have m^6^A-seq identified m^6^A peak and the m^6^A-LAIC-seq data show that the full-length RNAs are mostly in m^6^A negative fraction. In contrast, m^6^A-seq identified three m^6^A peaks on its pseudogene *DSTNP2*, and the m^6^A-LAIC-seq data showed that its full-length RNAs were greatly enriched in m^6^A positive fraction (Fig. 1e). Similar results were also observed for other pairs of protein-coding genes and pseudogenes in GM12878 (Fig. 1e) as well as H1 cell lines (Additional file 1: Figure S3d). The above results suggest that the m^6^A levels of pseudogenes are increased on novel sites, indicating de novo formation of m^6^A motifs on pseudogenes. As shown in Additional file 1: Figure S3e, there are pseudogene-specific m^6^A motifs in the pseudogene-specific peaks of *DSTNP2* and *NAP1L4P1*, suggesting that mutations can generate novel m^6^A motifs on pseudogenes.

### Convergent evidence support the m^6^A motifs on pseudogenes evolved under positive natural selection

**Fig. 2:**
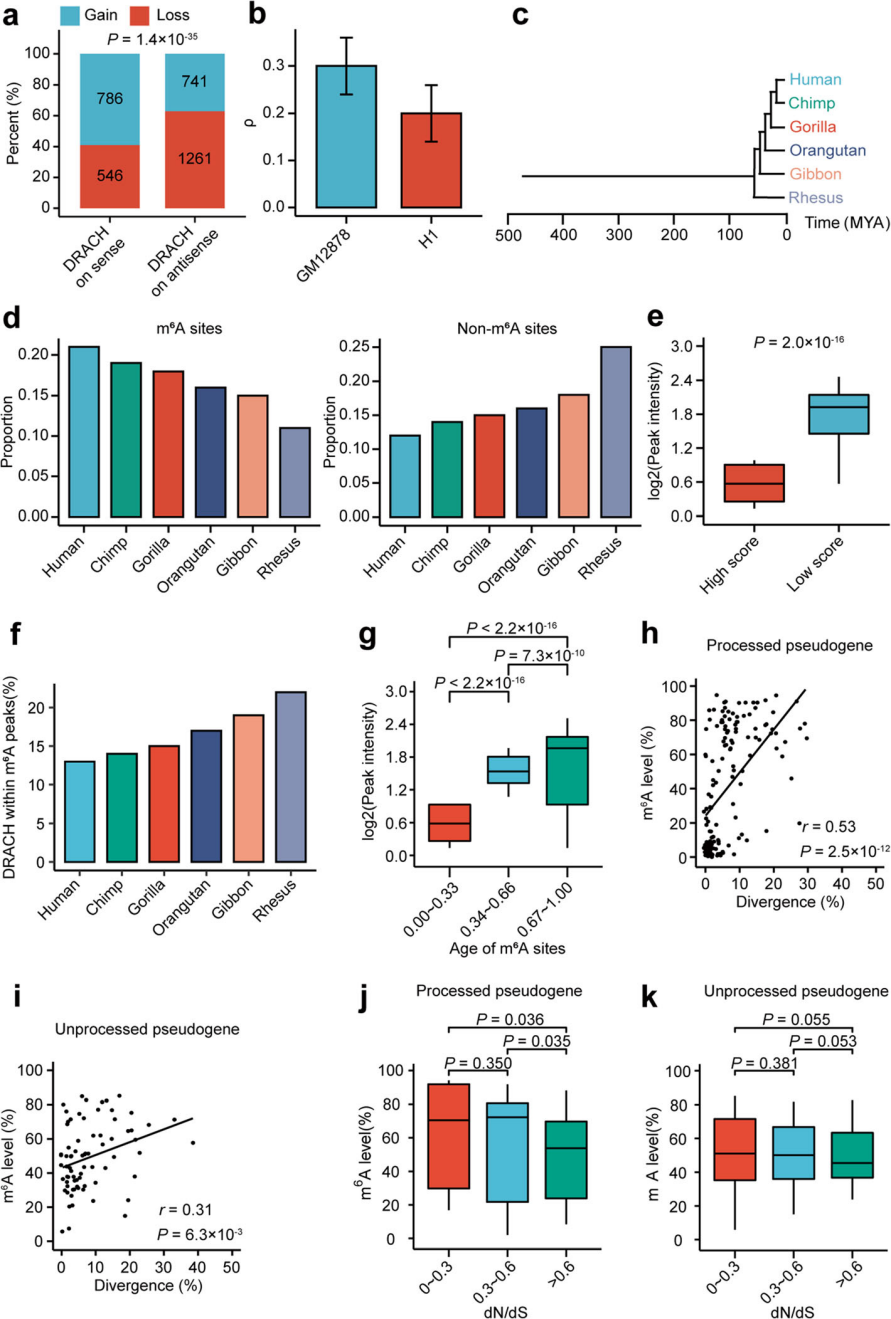
Positive natural selection of the m^6^A on pseudogenes in GM12878 cell line. **a** Stacked barplot comparing the proportions of pseudogenes gaining/losing m^6^A motif (DRACH) on sense and antisense strands respectively. **b** Bar plot showing the estimates of ρ for DRACH m^6^A motifs for GM12878 and H1 cells respectively. Error bars represent standard errors. **c** A schematic phylogeny of the species studied for age determination. **d** The distributions of the ages of m^6^A sites (left) and control non-m^6^A DRACH sites (right). The age of each site was based on the most distantly related species in which the site was conserved. **e** Box plot comparing the peak intensities between m^6^A sites with high (top 25%, strong constraint) and low (bottom 25%, weak constraint) rejected substitution scores. **f** Barplot showing the percentages of DRACH motifs within m^6^A peaks of GM12878 cells for DRACH motif of different ages. **g** Box plot comparing the peak intensities of m^6^A sites with different normalized ages (1 represents the oldest, while 0 represents the youngest) of pseudogenes. **h**, **i** Scatter plots showing the correlation of the sequence divergences between pseudogenes and their cognate protein-coding genes with the m^6^A levels of pseudogenes for processed pseudogenes (**h**) and unprocessed pseudogene (**i**), respectively. **j**, **k** Box plot comparing m^6^A level of pseudogenes with different ranges of dN/dS ratios of their cognate coding genes for processed (**j**) and unprocessed (**k**) pseudogenes, respectively

If de novo formation of m^6^A motifs on pseudogenes is under positive natural selection, we would expect to see a higher probability of obtaining novel m^6^A motifs than losing m^6^A motifs on pseudogenes in the evolution. To test this, we used the m^6^A motifs on the antisense strand as a neutral background, because of equal natural mutation probabilities on both strands. As shown in Fig. 2a, a significantly higher proportion of pseudogene on sense strand than on antisense strand has gained m^6^A motifs, supporting positive natural selection of the gained m^6^A motifs on pseudogenes (*P* = 1.4× 10^−35^, two-tailed chi-square test).

To further elucidate the evolutionary landscape of the m^6^A sites on pseudogenes, we took advantage of INSIGHT (Inference of Natural Selection from Interspersed Genomically coHerent elemenTs) to estimate ρ, which represents the proportion of negatively selected m^6^A sites on pseudogenes. Our estimate of ρ of the m^6^A sites on pseudogenes was 0.2 ~ 0.3 (Fig. 2b), lower than the previously estimated ρ of 0.33~0.56 for the m^6^A sites on 3′UTRs of mRNAs [33], suggesting that the m^6^A on pseudogenes are less conserved. In addition, the estimated ages of the m^6^A sites, based on a phylogenetic tree of human and representative non-human primates (Fig. 2c), were much younger than the non-m^6^A “DRACH motifs” on pseudogenes, suggesting that the birth rate of m^6^A sites is faster than random drift in primates evolution (Fig. 2d). We then calculated the rejected substitution scores, which measure the nucleotide-level constraint [34], to study the relationship between m^6^A and site conservation. We found that the less conserved m^6^A sites had significantly higher m^6^A peak intensities than conserved m^6^A sites (Fig. 2e). These results are consistent with our finding that there is a selection pressure of gaining new m^6^A motifs on pseudogenes, further supporting that m^6^A sites on pseudogenes are positively selected.

We were then interested in whether evolutionarily older pseudogenes tended to have stronger m^6^A modification. Based on the above calculated ages of these m^6^A sites, we found that the older the “DRACH” motifs are, the higher proportions of them are within detectable m^6^A peaks in GM12878 cells (Fig. 2f). In contrast to that only 13% of human-specific “DRACH” motifs were detected in m^6^A peaks in GM12878 cells, 22% of human and rhesus shared “DRACH” motifs were within m^6^A peaks detected in the same cell line. Consistently, we found a significant positive correlation between ages and intensities of m^6^A peaks (Fig. 2g). These results suggest that generating “DRACH” motifs works as the critical first step, followed by long evolution for them to get strong m^6^A methylation. In addition, we found a significant positive correlation between the sequence divergences and m^6^A levels for processed pseudogenes (*P* = 2.5× 10^−12^, Pearson correlation) and unprocessed pseudogenes (*P* = 0.006, Pearson correlation) respectively (Fig. 2h, i). Considering that the peak intensities of m^6^A peaks are overall conserved between human and mouse [35], the above results support that pseudogenes especially processed pseudogenes were originally methylated at a low level but evolved to have high methylation level quickly. Interestingly, highly m^6^A methylated pseudogenes, especially processed pseudogenes, tend to have cognate protein-coding genes with lower dN/dS ratios (ratio of nonsynonymous substitution rate to synonymous substitution rate), which represent the genes with higher essentiality and stronger functional constraint in the evolution, suggesting that important genes are more likely regulated by the modification of their processed pseudogene transcripts (Fig. 2j, k). On the other hand, we found the highly m^6^A methylated processed pseudogenes tend to have cognate protein-coding genes with shorter 5′UTRs, longer CDSs and 3′UTRs, lower GC contents, suggesting that these gene features of cognate coding genes may also relate to the m^6^A evolution of processed pseudogenes (Additional file 1: Figure S4a-d). Similar results were observed in H1 cells (Additional file 1: Figure S5a-l).

### m^6^A facilitates the cytosolic degradation of processed pseudogenes

**Fig. 3:**
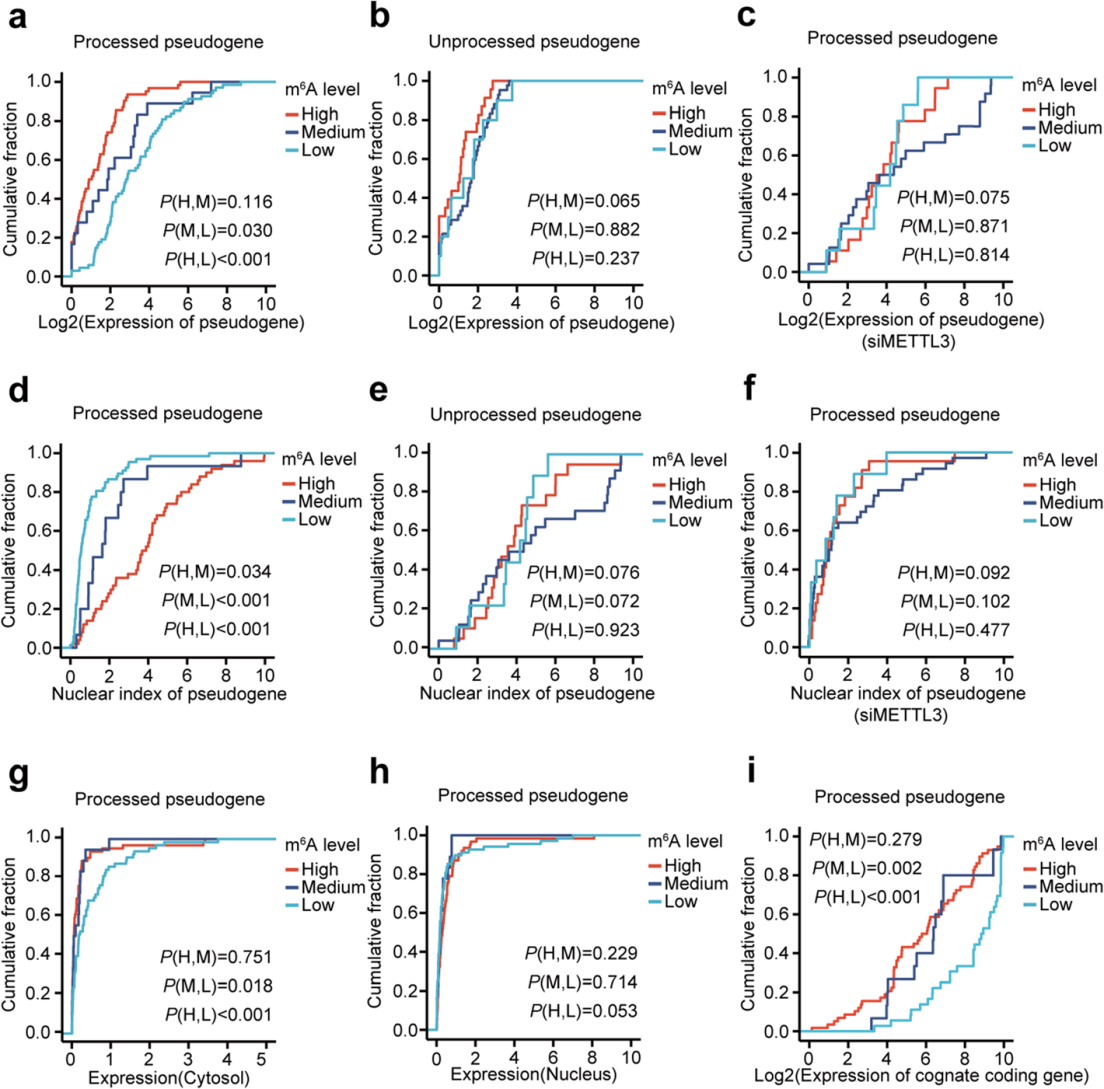
m^6^A facilitates the cytosolic degradation of processed pseudogenes in the GM12878 cell line. **a**, **b** Plot of cumulative fraction of expression for processed (**a**) and unprocessed (**b**) pseudogene with high (m^6^A level ≥ 0.6), medium (0.6 > m^6^A level ≥ 0.3), and low (m^6^A level < 0.3) m^6^A levels, respectively. Two-tailed Wilcoxon *P* values are indicated at the bottom right corner. **c** Plot of cumulative fraction of expression for processed pseudogenes with high (m^6^A level ≥ 0.6), medium (0.6 > m^6^A level ≥ 0.3), and low (m^6^A level < 0.3) m^6^A levels respectively in *METTL3* knockdown cells. Two-tailed Wilcoxon *P* values are indicated at the bottom right corner. **d**, **e** Plot of cumulative fraction of nuclear indexes (ratio of expression between the nucleus and cytosol) for processed (**d**) and unprocessed (**e**) pseudogenes with high, medium, and low m^6^A levels, respectively. Two-tailed Wilcoxon *P* values are indicated at the bottom right corner. **f** Plot of cumulative fraction of nuclear indexes for processed pseudogenes with high, medium, and low m^6^A levels respectively in *METTL3* knockdown cells. Two-tailed Wilcoxon *P* values are indicated at the bottom right corner. **g**, **h** Plot of cumulative fraction of cytosolic (**g**) and nuclear (**h**) gene expression for processed pseudogene with high, medium, and low m^6^A levels respectively. Two-tailed Wilcoxon *P* values are indicated at the bottom right corner. **i** Plot of cumulative fraction of expression of cognate protein-coding genes for processed pseudogene with high, medium, and low m^6^A levels respectively. Two-tailed Wilcoxon *P* values are indicated at the top left corner and bottom right corner, respectively

In order to understand the evolutionary pressure for pseudogenes to become highly methylated, we explored the functional consequences of m^6^A methylation on pseudogenes. m^6^A has been reported to promote nuclear export [24] and cytosolic degradation [21–23], we first tested whether m^6^A affected the gene expression of pseudogenes using the input of m^6^A-LAIC-seq data. We found the highly methylated (m^6^A level ≥ 0.6) (*P* < 0.001, two-tailed Wilcoxon test) and moderately methylated (0.6 > m^6^A level ≥ 0.3) (*P* = 0.030, two-tailed Wilcoxon test) processed pseudogenes had dramatically lower gene expression than lowly methylated (m^6^A level < 0.3) processed pseudogenes (Fig. 3a) in GM12878 cells, suggesting that m^6^A promotes the degradation of processed pseudogenes. Nevertheless, we did not observe a similar result for unprocessed pseudogenes (Fig. 3b), suggesting that the m^6^A-induced cytosolic degradation might be a specific function to processed pseudogenes. To further confirm whether different expression of processed pseudogenes is due to m^6^A, we sequenced the input, cytoplasmic, and nuclear RNAs of control and METTL3-knockdown GM12878 cells. Expression differences among the processed pseudogenes with different categories of m^6^A levels were observed in control cells (Additional file 1: Figure S6a). However, the differences became not significant in METTL3-knockdown cells, indicating the expression differences of processed pseudogenes are dependent on m^6^A (Fig. 3c).

To test whether m^6^A facilitates the nuclear export of pseudogene RNAs, we took advantage of the CSHL RNA-seq of separated cytosol and nucleus RNAs in GM12878 and H1 cell lines [36]. We found the highly methylated processed pseudogenes had dramatically higher nuclear indexes (ratio of expression between nucleus and cytosol) than moderately methylated (*P* = 0.034, two-tailed Wilcoxon test) and lowly methylated processed pseudogenes (*P* < 0.001, two-tailed Wilcoxon test) (Fig. 3d) in GM12878 cell line, suggesting stronger nuclear retention or cytosolic depletion of m^6^A methylated processed pseudogenes. We did not observe a similar result for unprocessed pseudogenes (Fig. 3e), which is consistent with the above result that the m^6^A of processed other than unprocessed genes is negatively correlated with gene expression (Fig. 3a, b). Similar results for processed pseudogenes were also observed in control GM12878 cells (Additional file 1: Figure S6b), but the differences were not significant in METTL3-knockdown cells, indicating that the nuclear index differences of processed pseudogenes are due to m^6^A differences (Fig. 3f). Because m^6^A has been reported to promote the nuclear export other than nuclear retention of RNAs [21], m^6^A likely facilitates the cytosolic degradation of processed pseudogenes. We then found the m^6^A of processed pseudogenes were negatively correlated with the gene expression in cytosol (Fig. 3g), but not significant with the gene expression in nucleus (Fig. 3h), strongly supporting the role of m^6^A on degrading the processed pseudogenes in cytosol.

Interestingly, we also found that the m^6^A levels of processed other than unprocessed pseudogenes were negatively correlated with the gene expression of their cognate protein-coding genes (Fig. 3i; Additional file 1: Figure S6c) and positively correlated with the nuclear indexes of their cognate protein-coding genes (Additional file 1: Figure S6d, e), suggesting that the m^6^A on the RNAs of pseudogenes may affect mRNA expression of their cognate protein-coding genes. Similar results were observed in H1 cells (Additional file 1: Figures S7a-f; S8a-d).

### m^6^A of processed pseudogenes disrupt their crosstalk with their cognate protein-coding genes

**Fig. 4:**
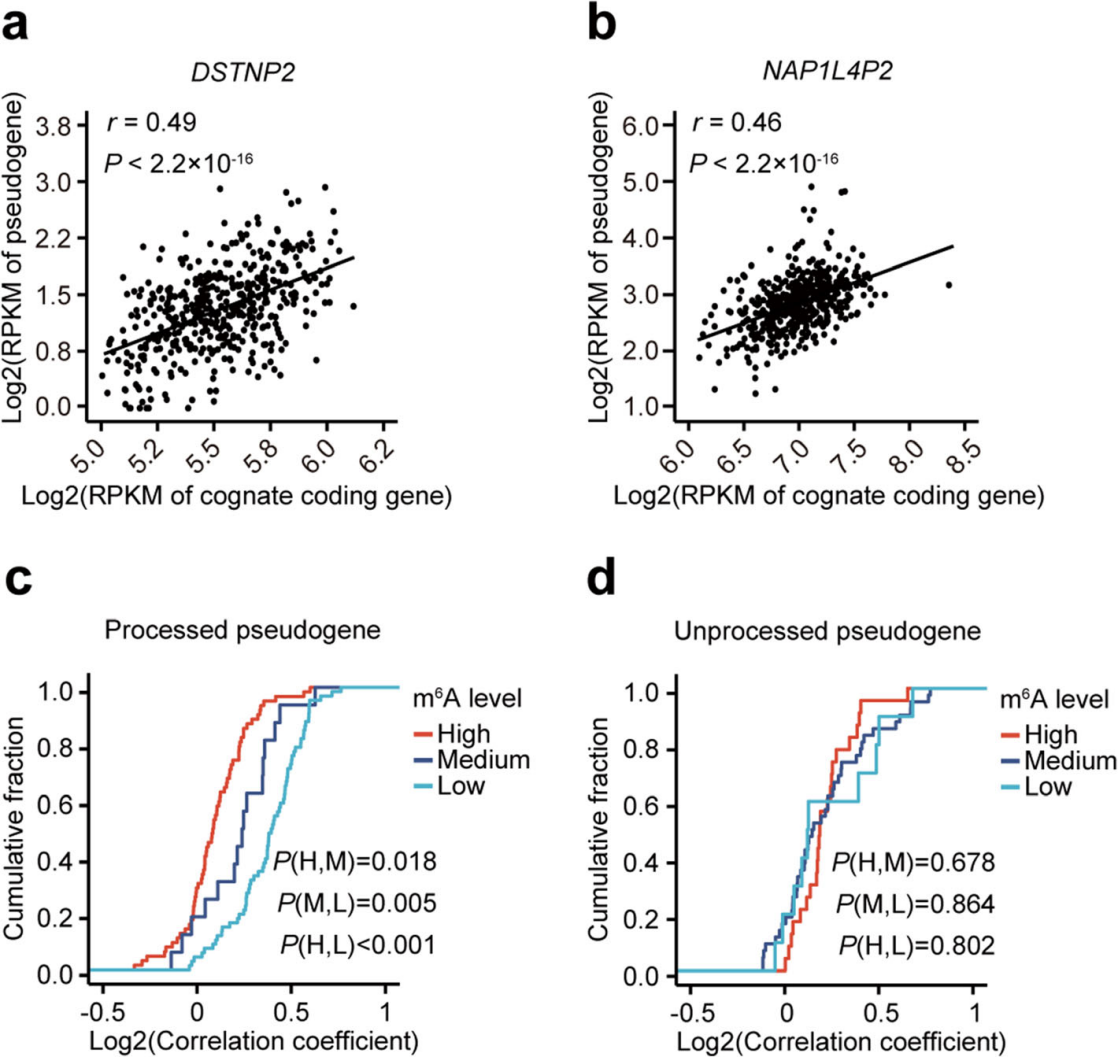
m^6^A of processed pseudogenes disrupt their crosstalk with their cognate protein-coding genes. **a**, **b** Scatter plots showing the correlation of the expressions of two representative processed pseudogenes *DSTNP2* (**a**) and *NAP1L4P2* (**b**) with their cognate protein-coding genes among 462 B-lymphoblastoid cell lines. *P* values of Pearson correlations are indicated at the top left corners. **c**, **d** Plot of cumulative fraction of correlation coefficients of gene expressions for processed (**c**) and unprocessed (**d**) pseudogenes with their cognate protein-coding genes among 462 B-lymphoblastoid cell lines. Pseudogenes with high, medium, and low m^6^A levels are shown separately. Two-tailed Wilcoxon *P* values are indicated at the bottom right corners

To further address whether m^6^A of pseudogenes affects the crosstalk between pseudogenes and their cognate protein-coding genes, we analyzed the RNA-seq data of B-lymphoblastoid cell lines (BLCL), the same cell type as GM12878, from 462 European participants of 1000 genome project [37, 38]. We found that 164 out of 223 pairs of pseudogenes and cognate protein-coding genes showed significant correlations (FDR < 0.05) of gene expressions, 74% of them are positive correlations, which is consistent with the positive regulatory roles of ceRNAs (Examples of positive correlations are shown in Fig. 4a, b).

To test whether the m^6^A of pseudogenes affects the crosstalk between pseudogenes and their cognate protein-coding genes, we compared the correlation coefficients of the gene expressions between pseudogenes and their cognate protein-coding genes in three categories of gene pairs with different m^6^A levels of pseudogenes. We found that the processed pseudogenes with higher m^6^A levels had significantly lower correlation coefficients (*P* = 0.018, two-tailed Wilcoxon test) (Fig. 4c), suggesting that m^6^A of processed pseudogenes disrupt their crosstalk with their cognate protein-coding genes. However, we did not observe a similar result for unprocessed pseudogenes (Fig. 4d).

### Experimental validation of the m^6^A on two processed pseudogenes reduce their crosstalk with their cognate protein-coding genes *via* promoting the decay of pseudogenes

To experimentally test whether the m^6^A of pseudogenes affects the expression of their cognate protein-coding genes, we selected two representative processed pseudogenes *DSTNP2* and *NAP1L4P1* for further validation in GM12878 cell line.

**Fig. 5:**
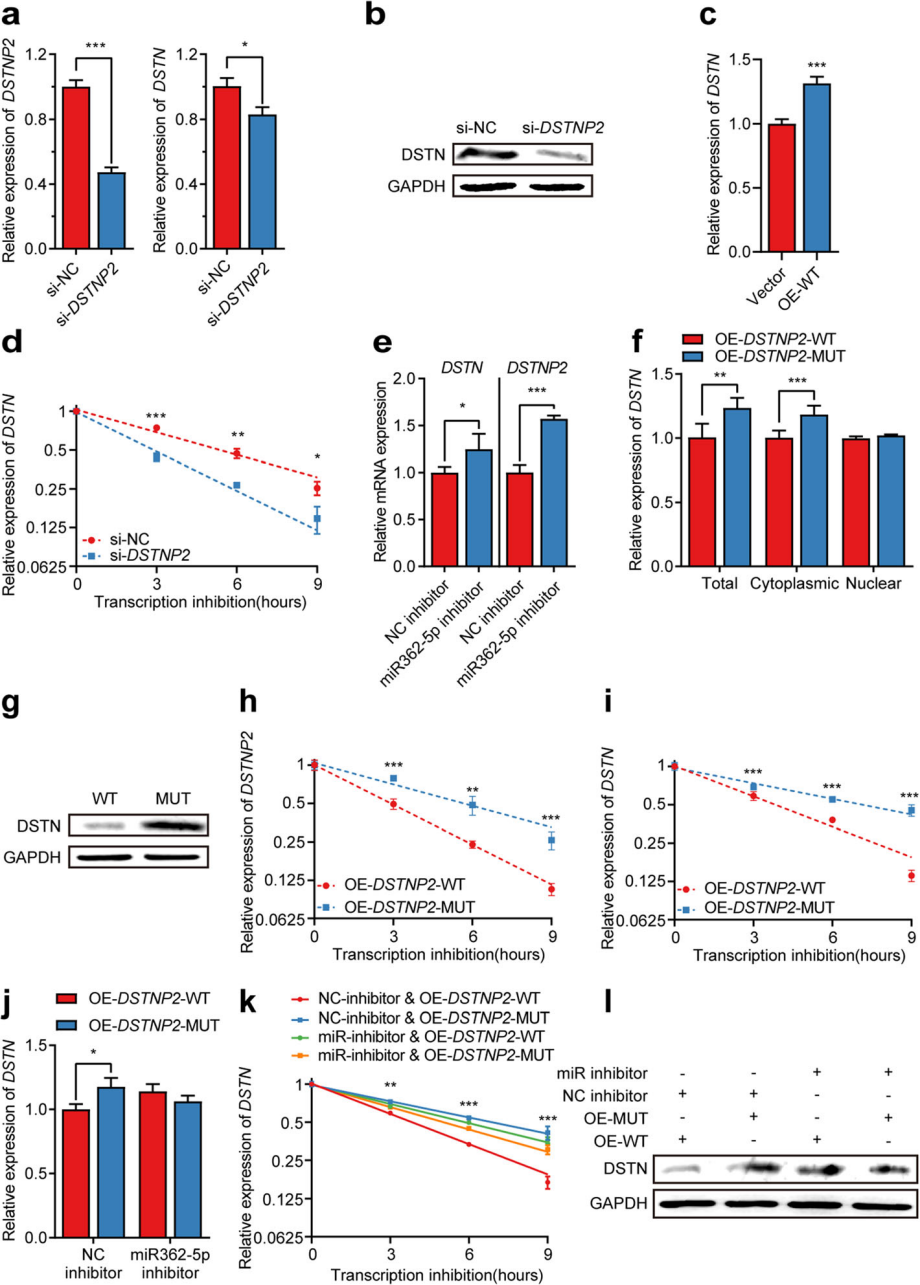
Experimental validation of m^6^A on processed pseudogene *DSTNP2* affects the expression of *DSTN* in GM12878 cell line. **a** Relative gene expression of *DSTNP2* and *DSTN* after *DSTNP2* knockdown using siRNA. * *P*< 0.05; *** *P*< 0.001 (two-tailed t test). **b** Western blot comparing the protein levels of DSTN in control and *DSTNP2* knockdown cells. **c** The relative expression of *DSTN* upon overexpression empty vector and wild type *DSTNP2* respectively. Error bars represent standard errors. *** *P*< 0.001 (two-tailed t test). **d** The relative expression of *DSTN* at different time points after transcription inhibition in control and *DSTNP2* knockdown cells respectively. Error bars represent standard errors. * *P*< 0.05; ** *P*< 0.01; *** *P*< 0.001 (two-tailed t test). **e** The relative expression of *DSTN* and *DSTNP2* after adding control inhibitor versus miR362-5p inhibitor. **f** Bar plot comparing the relative expression of total, cytoplasmic and nuclear *DSTN* respectively upon overexpression of wild type versus m^6^A sites-mutated *DSTNP2*. **g** Western blot comparing the protein levels of DSTN upon overexpression of wild type *DSTNP2* versus m^6^A site-mutated *DSTNP2*. **h**, **i** The relative expression of *DSTNP2* (**h**) and *DSTN* (**i**) upon overexpression of wild type *DSTNP2* versus m^6^A site-mutated *DSTNP2* at different time points after transcription inhibition. Error bars represent standard errors. ** *P* < 0.01; *** *P* < 0.001 (two-tailed t test). **j** The relative expression of *DSTN* upon overexpression of wild-type *DSTNP2* versus m^6^A site-mutated *DSTNP2* after adding control inhibitor and miR362-5p inhibitor respectively. Error bars represent standard errors. * *P* < 0.05 (two-tailed t test). **k** The relative expression of *DSTN* at different time points after transcription inhibition upon overexpression of wild-type *DSTNP2* versus m^6^A site-mutated *DSTNP2* after adding control inhibitor and miR362-5p inhibitor respectively. Error bars represent standard errors. ** *P*< 0.01; *** *P*< 0.001. l Western blot showing the protein level of *DSTN* upon overexpression of wild type *DSTNP2* versus m^6^A site-mutated *DSTNP2* after adding control inhibitor and miR362-5p inhibitor, respectively

First, we tested whether *DSTNP2* can regulate its protein-coding cognate *DSTN*. We found knockdown of *DSTNP2* by siRNA significantly down-regulated mRNA as well as the protein expression of DSTN (Fig. 5a, b; Additional file 1: Figure S10a), while overexpression of *DSTNP2* significantly upregulated the expression of *DSTN* (Fig. 5c), consistent with our observation that their gene expressions were positively correlated in a population of B-lymphoblastoid cell lines (Fig. 4a). In order to test whether *DSTNP2* modules *DSTN* *via* ceRNA mechanism, we first confirmed that knockdown of *DSTNP2* could promote the degradation of *DSTN* (Fig. 5d). We then predicted the miRNA binding sites and found 6 miRNAs potentially targeting both *DSTNP2* and *DSTN* (Additional file 1: Figure S9a). We selected miR-362-5p for experimental validation due to their higher expression level according to ENCODE miRNA-seq data of GM12878 [39]. We found inhibition of miR-362-5p significantly increased the expression of both *DSTNP2* and *DSTN*, indicating that miR-362-5p targets both *DSTNP2* and *DSTN* (Fig. 5e).

To test whether the m^6^A of *DSTNP2* affects the crosstalk between *DSTNP2* and *DSTN*, we mutated all the 8 m^6^A sites of *DSTNP2* without disrupting the binding sites of miR-362-5p (Additional file 1: Figure S9b), and then overexpressed the wild type and mutant respectively into GM12878 cell line. Compared with wild-type *DSTNP2*, we found overexpressing *DSTNP2* mutant resulted in significantly higher mRNA as well as protein expression of *DSTN* (Fig. 5f, g; Additional file 1: Figure S10b). We then separated the cytosolic and nuclear RNAs and found the mutations of *DSTNP2* m^6^A sites specifically affected the expression of cytosolic RNAs of *DSTN* (Fig. 5f). To test whether the m^6^A of *DSTNP2* affects the stabilities of cytoplasmic RNAs, we measured the degradation rates of *DSTNP2* and *DSTN* RNAs. We found that *DSTNP2* mutant had significantly higher stability than wild type *DSTNP2* (Fig. 5h), and overexpression of *DSTNP2* mutant resulted in significantly higher stability of its cognate protein-coding gene *DSTN* (Fig. 5i; Additional file 1: Figure S10c), suggesting that the m^6^A of *DSTNP2* promotes the degradation of *DSTNP2* and indirectly decreases the stability of *DSTN*. When miR-362-5p was inhibited, overexpression of wild-type and mutant *DSTNP2* did not show significantly different effects on the gene expression, RNA stability, and protein expression of *DSTN*, suggesting that m^6^A mediated degradation of *DSTNP2* reduced the ceRNA effects of *DSTNP2* on *DSTN* (Fig. 5j–l).

Similar results were observed for the other processed pseudogene *NAP1L4P1*. *NAP1L4P1* can also upregulate the expression of *NAP1L4* (Additional file 1: Figure S11a, b). Overexpression of *NAP1L4P1* m^6^A-mutant resulted in significantly higher cytoplasmic abundance as well as stability of its cognate protein-coding gene *NAP1L4* (Additional file 1: Figure S11c-e), suggesting that the m^6^A of *NAP1L4P1* promotes the degradation of *NAP1L4P1* and indirectly decreases the stability of *NAP1L4*. However, we did not succeed in identifying the miRNA that mediates this process.

## Discussion

In this study, we found convergent evidences supporting the adaptive accumulation of m^6^A sites on human pseudogenes through mutations in the evolution, resulted in higher m^6^A levels on the RNAs of pseudogenes. Through integrating with public dataset, we realized the m^6^A on the RNAs of processed other than unprocessed pseudogenes promoted cytosolic RNA degradation and reduced their regulatory effects on their cognate mRNAs mediated by ceRNA mechanism. Our discovery revealed a novel evolutionary role of m^6^A in cleaning up the unnecessary RNAs of processed pseudogenes to attenuate the interrupting of the regulatory pathway of miRNA on mRNAs. The novel finding in this study also unveiled a mystery of how cells clean transcribed processed pseudogenes through splicing-independent mechanisms.

In general, pseudogenes are nonfunctional, and evolutionarily neural, K_A_/K_S_ test of pseudogenes indicates low functional constraint of their protein-coding ability [40]. However, carrying a piece of useless DNA is deleterious because it costs energy, there is a tendency of losing pseudogenes in the evolution and the existing pseudogenes tend to be young [41, 42]. On the other hand, transcription of pseudogenes may not be as neutral as previously thought either. It was reported that only half of human transcribed pseudogenes were conserved in rhesus macaque and only 3% of them were conserved in mouse [43], indicating a trend of rapidly losing transcribed pseudogenes in the evolutionary history. Because the sequences of pseudogenes are quite similar as their cognate protein-coding genes, RNA binding proteins and miRNAs may not be able to distinguish them, therefore the transcribed pseudogenes may interrupt the existing regulatory network through mechanisms such as competitive endogenous RNAs [44]. Since most transcribed pseudogenes are evolutionarily young, they are not likely to have obtained important functions, most of these interruptions should be deleterious and degradation of them might be adaptive. For these transcribed pseudogenes, splicing dependent NMD mechanism can recognize the unprocessed pseudogenes and trigger the RNA degradation in the cytoplasm. However, processed pseudogenes cannot degrade through the canonical NMD pathway due to the lack of splicing. In this study, we found that the processed pseudogenes took advantage of the m^6^A-mediated cytosolic RNA degradation mechanism to get rid of these interrupting RNAs in the evolution. We also found that these methylated m^6^A motifs were evolutionarily younger than those unmethylated m^6^A motifs, and there was a specific selective pressure to obtain m^6^A motif on these pseudogenes, indicating positive selection of m^6^A on the transcribed pseudogenes.

Though these competitive endogenous RNAs transcribed from pseudogenes are deleterious at the early stage of evolution, it is also possible that some of them become functionally important. For example, the lncRNA *Oct4P4* plays an important role in inducing and maintaining the silencing of the ancestral *Oct4* gene in differentiating mouse embryonic stem cells (mESCs) [45]. The cellular 5S rRNA pseudogene transcripts, which are unshielded following depletion of their respective binding proteins by the virus, induces RIG-I-mediated antiviral immunity [46]. The *TUSC2P* (tumor suppressor candidate-2 pseudogenes) promotes *TUSC2* function by binding multiple microRNAs, and ectopic expression of *TUSC2P* and *TUSC2* inhibits cell proliferation, survival, migration, invasion, and colony formation, and increases tumor cell death [47]. Pseudogenes regulate mRNAs and lncRNAs *via* the piRNA pathway in the germline [48]. The Pan-Cancer analysis of pseudogene expression has showed that pseudogenes can be a new paradigm for investigating cancer mechanisms and discovering prognostic biomarkers [49]. In principle, there could be widespread pseudogenes that interfere with the regulatory networks during the long evolutionary history, and thus, important functions of pseudogenes could have the opportunity to get evolved.

Here, we found a novel mechanism of RNA surveillance for processed pseudogenes *via* m^6^A RNA methylation. How does this mechanism evolve? In this study, we found a significantly higher probability of obtaining novel m^6^A motifs on sense strand than antisense strand, strongly suggesting that obtaining m^6^A sites on pseudogenes tends to be adaptive and accumulating in the evolution. In this way, the elevated m^6^A on processed pseudogenes are the results of positively selected mutations that produce novel m^6^A sites. Alternatively, we cannot rule out that there is an unknown specific mechanism, such as RBP-mediated specific regulation of m^6^A [20, 50], to recognize processed pseudogenes and mark them for cytosolic degradation by m^6^A RNA modification. However, whether and how the cells mark processed pseudogenes is still unknown and requires further investigation.

The roles of m^6^A played in evolution especially human evolution are still elusive. Ma *et al*. reported that newly acquired m^6^A modifications in humans were under positive selection [51]. However, Liu *et al*. had contradictory viewpoint that these newly acquired m^6^A sites were neutral and most m^6^A sites in protein-coding regions were nonfunctional and nonadaptive [52]. Recently, Zhang *et al*. reported that a significant fraction of 3′ UTR m^6^A sites were under negative selection and recently gained 3′ UTR m^6^A in humans were positively selected [33]. Our study further indicates that m^6^A on human transcribed pseudogenes are evolutionarily young and evolved under positive selection, indicating that m^6^A plays more important and widespread roles than expected in human evolution.

Promoting cytosolic RNA degradation is an important molecular role of m^6^A RNA modification, it plays important roles in diverse physiology process which need to remove existing RNAs. It removes the RNAs of current cell status to facilitate cell fate transition [35]; it cleans the maternal RNAs during the maternal-to-zygotic transition [53]. Removing harmful transcribed pseudogenes is a novel role of m^6^A mediated RNA decay. In addition, nonsense mutations and frame-shift mutations that occur on intronless protein-coding genes can also produce poisonous truncated proteins, and we found the RNAs of protein-coding genes with single exons and the limited number of exons also showed significantly higher m^6^A levels than genes with a larger number of exons, suggesting that m^6^A-mediated RNA degradation may also play roles for RNA surveillance of protein-coding genes with single or a few of exons (Additional file 1: Figure S12a-d).

## Conclusions

Our discovery reveals a novel evolutionary role of m^6^A RNA modification in cleaning up the unnecessary processed pseudogene transcripts to attenuate their interfering with the regulatory network of mRNAs. It provides novel aspect on the importances of m^6^A in the evolution.

## Methods

### Data sources

The list of protein-coding genes and corresponding pseudogenes were compiled from GENCODE, Vega, and Pseudogene.org databases [54]. As described, the annotated pseudogenes must have at least one of the disablements, including premature stop codon, frame-shift, truncation at 5′ or 3′ end of CDS, deletion of an internal portion of CDS, and lack of locus-specific transcriptional evidence for process pseudogene [55]. The SNPs of GM12878 were obtained from Genome in a Bottle (GIAB) Consortium [56]. The raw data of nucleus and cytosol RNA-seq of GM12878 and H1 were obtained from ENCODE project [36]. The RNA-seq data of the lymphoblastoid cell lines of 462 individuals from the 1000 Genomes Project were obtained from the Geuvadis project [57]. The m^6^A-LAIC-seq data of GM12878 and H1 hESC cell lines were obtained from our previous publication [28]. The m^6^A-seq data of GM12878 cell line [32] and H1 hESC [35] were also obtained from previous publications. The ratio of dN/dS and GC contents were obtained from BioMart database [58]. miRNA binding targets on pseudogenes were obtained from the dreamBase database [59]. miRNA binding targets to protein-coding genes were obtained from the miRWalk database [60]. The microRNA-seq data of GM12878 cells were obtained from ENCODE project [39] (GEO accession: GSE143080).

### Processing of sequencing data

The RNA-seq, m^6^A-LAIC-seq, and m^6^A-seq reads were aligned to hg19 human genome using Hisat2 [29], known SNPs from the dbSNP database (GM12878 SNPs only for GM12878 data) were provided, and the mismatches at SNP loci were tolerated in the mapping. Only the reads with perfect match except for mismatches at SNPs were allowed. The proper paired and uniquely mapped reads were used for the downstream analyses. To evaluate the accuracy of this alignment procedure, we compared it with the conventional alignment procedure with default parameters of Hisat2.

### Gene expression analyses

RPKMs of genes were calculated using StringTie2 [61]. The raw data of nucleus and cytosol RNA-seq of GM12878 and H1 were remapped using the above reprocessing procedure, the RPKMs of nucleus and cytosol were normalized as described in the original paper [36], then the nuclear indexes were calculated as the ratio between normalized RPKMs of nucleus and cytosol.

### m^6^A analyses

We recalculated the m^6^A levels of all annotated genes including the compiled pseudogenes based on the reprocessed m^6^A-LAIC-seq data of GM12878 and H1 cells according to the method described in our previously published paper [28]. The m^6^A peaks were identified based on the reprocessed m^6^A-seq data of GM12878 [32] and H1 [35] cells according to our previously described method [35]. The single-nucleotide m^6^A sites were determined by combining the m^6^A sites predicted by sequence-based m^6^A site predictors SRAMP [62] and Whistle [63] within m^6^A peaks regions. The longest transcript of each gene was used in the analyses of gene features.

### Evolution analyses

To compare the m^6^A motifs between pseudogenes and their cognate protein-coding genes, we aligned the pseudogenes and their cognate protein-coding genes using ClustalW2 [64] with default parameters. The DRACH m^6^A motifs on pseudogenes and their cognate protein-coding genes were counted respectively within the aligned regions covered by m^6^A peaks of either pseudogenes or their cognate protein-coding genes. The DRACH motifs on the antisense strand were counted in the same way as the background.

The SRAMP [62] and Whistle [63] predicted single-nucleotide m^6^A sites within m^6^A peaks of pseudogenes were used to study the natural selection of m^6^A sites on pseudogenes. We used INSIGHT (Inference of Natural Selection from Interspersed Genomically coHerent elemenTs) [65] to estimate the proportion of m^6^A sites that are under negative selection. The rejected substitution scores calculated by GERP++ [66] were used to measure the nucleotide-level functional constraint of all m^6^A sites. The ages of individual m^6^A sites were determined using a phylostratigraphy approach [67] with pairwise alignments downloaded from the UCSC genome browser, the DRACH motifs outside the m^6^A peaks on pseudogenes were used as evolutionally neutral control sites.

### Cell culture, lentiviral production, and transduction

HEK293T (ATCC® CRL-3216™) cells were cultured in high glucose Dulbecco’s modified Eagle’s medium (Corning), supplemented with 10% FBS (Biological Industries) at 37 °C with 5% CO2. GM12878 cells were cultured in Roswell Park Memorial Institute Medium 1640 (Corning), supplemented with 15% FBS (Biological Industries) at 37 °C with 5% CO_2_. Both cell lines were obtained from GuangZhou Jennio Biotech Co., Ltd., authenticated and tested for the absence of mycoplasma contamination using Myco-Blue Mycoplasma Detector (Vazyme).

For lentiviral production, 293 T cells were seeded in 6 cm cell culture plates, and 24 hours later, cells were transfected with 6.4 μg of lentiviral backbone, 4.8 μg of psPAX2 (Addgene #12260), and 1.6 μg of pMD2.G (Addgene #12259) using LipoFiter reagent (HANBIO). Lentiviral supernatants were harvested at 48 h and 72 h after transfection and filtered through using a 0.45μm PVDF filter (Millipore) and concentrated using PEG. 5 × 10^4^ GM12878 cells were seeded in a TC-untreated plate and transduced with viral supernatants in the presence of polybrene (8 μg/μL). Twenty-four hours after transduction, cells were selected with puromycin (2 μg/mL).

### Construction of plasmid DNA

Wild type and m^6^A motif mutant DNA sequence of *DSTNP2* and *NAP1L4P1* genes were synthesized by GENEWIZ company. Lentiviral expression plasmids were generated using ClonExpress II One Step Cloning Kit (Vazyme, C112), by combining PCR-amplified cDNA and EcoR I/BamH I digested pCDH-CMV-MCS-EF1α-CopGFP-T2A-Puro (SBI) backbone.

### miRNA inhibitor and siRNA transfection

Cells were seeded in TC-untreated plates and transfected with specific miRNA inhibitors and inhibitor NC or siNC and specific siRNA using LipoFiter reagent (HANBIO). RNA samples and protein samples were harvested at 72 h after transfection for qRT-PCR.

### Immunoblotting

Proteins were extracted from cells by incubating with RIPA buffer (Cell Signaling Technology, Cat. 9806) on ice for 10 min and insoluble fraction was removed by centrifugation. Twenty micrograms of extracted protein was separated on 15% SDS-PAGE and transferred to PVDF membrane. Membranes were blocked in 5% BSA in Tris-Buffered Saline with 0.01% Tween 20 (TBS-T) at room temperature for 1 h and incubated overnight with primary antibodies diluted in 1% BSA/TBS-T at 4 °C, followed by incubating with HRP conjugated secondary antibody diluted in TBS-T for 1 h at room temperature, and visualized using Clarity™ Western ECL Substrate (Bio-Rad). The following antibodies were used for immunoblotting: DSTN (1:1000, Abcam, ab192262), GAPDH (1:1000, ab8245).

### RNA extraction and real-time quantitative PCR (qPCR)

Total RNA was extracted using the NucleoZol RNA reagent (MACHEREY-NAGEL). And fraction RNA was separated using the Cytoplasmic and Nuclear RNA Purification Kit (NORGEN). One microgram of DNA-free RNA was then reverse-transcribed using HiScript III RT SuperMix for qPCR (+gDNA wiper) (Vazyme, R232). qPCR was carried out using the ChamQ Universal SYBR qPCR Master Mix (Vazyme, Q711) and performed in an LC480 Real-Time PCR System (Roche). Fold-change was calculated using the 2^-∆∆CT^ method. The primers of pseudogenes and their cognate protein-coding genes, which had been appended in Additional file 2: Table S4, were designed at the places with sequence divergences (Additional file 1: Figure S8d).

### RNA sequencing

RNA-seq libraries of the input, cytoplasmic, and nuclear RNAs of GM12878 with and without METTL3 knockdown were prepared using the VAHTS® mRNA-seq V2 Library Prep Kit for Illumina from Vazyme and sequenced on Illumina HiSeq 2500 platform to generate 150 bp paired-end reads.

### RNA stability assay

The final concentration of 5 μg/mL Actinomycin D (Sigma, A9415) was added to cells to assess RNA stability. After incubation for indicated time points, the cells were collected, and RNA samples were extracted for reverse transcription and qPCR. *18S* was used as the reference gene and fold-change was calculated using the 2^-∆∆CT^ method.

## Supplementary Information

**Additional file 1: Figure S1.** The optimization of conventional HISAT2 mapping procedure. **Figure S2.** Comparisons of m^6^A between pseudogenes and their cognate protein-coding genes in GM12878 and H1 cell line. **Figure S3.** Comparisons of m^6^A between pseudogenes and their cognate protein-coding genes in H1 cell line. **Figure S4.** Comparisons of pseudogenes m^6^A among 5′UTRs, CDSs, 3′UTRs and GC contents in their cognate protein-coding genes in GM12878 cell line. **Figure S5.** Positive natural selection of the m^6^A on pseudogenes in H1 cell line. **Figure S6.** m^6^A facilitates the cytosolic degradation of cognate protein-coding genes of processed pseudogenes in GM12878 cell line. **Figure S7.** m^6^A facilitates the cytosolic degradation of processed pseudogenes in H1 cell line. **Figure S8.** m^6^A facilitates the cytosolic degradation of cognate protein-coding genes of processed pseudogenes in H1 cell line. **Figure S9.** The gene regulatory network and primer design principles of processed pseudogenes and cognate protein-coding gene*s*. **Figure S10.** The uncropped bots of western blots in Fig. 5. **Figure S11.** Experimental validation of m^6^A on processed pseudogene *NAP1L4P1* affects the expression of *NAP1L4* in GM12878 cell line. **Figure S12.** Correlation between m^6^A levels and exon numbers of coding genes.

**Additional file 2: Table S1.** The list of pseudogenes and their cognate protein-coding genes. **Table S2.** The m^6^A levels of pseudogenes and their cognate protein-coding genes in GM12878 cell line. **Table S3.** The m^6^A levels of pseudogenes and their cognate protein-coding genes in H1 cell line. **Table S4.** The primers of processed pseudogenes (*DSTNP2*; *NAP1L4P1*) and their cognate protein-coding genes (*DSTN*; *NAP1L4*). **Table S5.** The list of collected GEO datasets. **Table S6.** The list of collected EBI datasets.

**Additional file 3.** Review history.

## Data Availability

The raw sequence data have been deposited in the GEO dataset under the accession number GSE172219 [68]. The accession numbers and links of third-party high-throughput sequencing data obtained from the GEO and EBI database were listed in Additional file 2: Tables S5 and S6, respectively.
