# Supplementary material for: Positive natural selection of N6-methyladenosine on the RNAs of processed pseudogenes
Source: Genome Biol. 2021 Jun 13;22:180. doi: 10.1186/s13059-021-02402-2 (PMC8201931; doi:10.1186/s13059-021-02402-2)

Additional file 1 : Fig. S1 to S12 for

Positive natural selection of N6-methyladenosine on the RNAs of processed pseudogenes

Additional Fig. S1. The optimization of conventional HISAT2 mapping procedure. a, b, c Scatter plot comparing the aligned counts between the conventional HISAT2 mapping procedure (HH) and the new HISAT2 mapping procedure (HHS) for whole genes **a**, pseudogenes **b** and cognate coding genes **c**, respectively. **d, e** UCSC genome browser tracks of m^6A-LAIC-seq input data between HH and HHS showing representative examples of pseudogenes and their cognate protein-coding genes. Read-coverage tracks of input fractions of m^6A-LAIC-seq shown along with re-assignment of reads (red for HHS and cyan for HH; re-assigned reads between HH and HHS are indicated by color). Plots showing the track and re-assignment of reads between HH and HHS for protein-coding gene RPS7 and corresponding pseudogene RPS7P1 **d** and protein-coding gene PPP1R14B and corresponding pseudogene PPP1R14BP3 **e**, respectively.

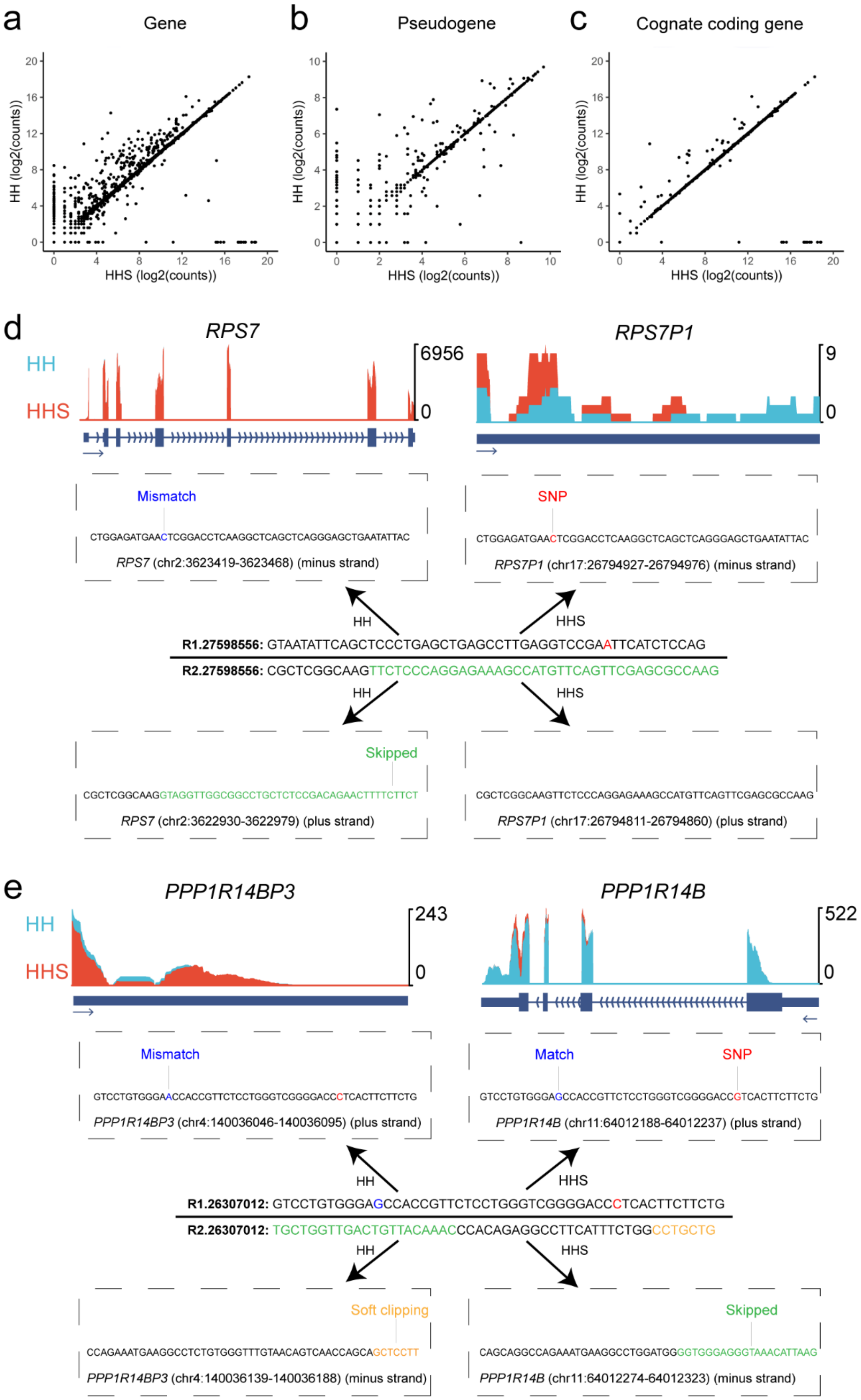

a
Gene
b
Pseudogene
c
Cognate coding gene
HH (log2(counts))
HHS (log2(counts))
d
RPS7
RPS7P1
HH
HHS
6956
0
9
0
Mismatch
CTGGAGATGAACTCGGACCTCAAGGCTCAGCTCAGGGAGCTGAATATTAC
RPS7 (chr2:3623419-3623468) (minus strand)
SNP
CTGGAGATGAACTCGGACCTCAAGGCTCAGCTCAGGGAGCTGAATATTAC
RPS7P1 (chr17:26794927-26794976) (minus strand)
R1.27598556: GTAATATTCAGCTCCCTGAGCTGAGCCTTGAGGTCCGAATTCATCTCCAG
R2.27598556: CGCTCGGCAAGTTCTCCCAGGAGAAAGCCATGTTCAGTTCGAGCGCCAAG
Skipped
CGCTCGGCAAGGTAGGTTGGCGGCCTGCTCTCCGACAGAACTTTTCTTCT
RPS7 (chr2:3622930-3622979) (plus strand)
CGCTCGGCAAGTTCTCCCAGGAGAAAGCCATGTTCAGTTCGAGCGCCAAG
RPS7P1 (chr17:26794811-26794860) (plus strand)
e
PPP1R14BP3
PPP1R14B
243
522
Mismatch
GTCCTGTGGGAACCACCGTTCTCCTGGGTCGGGGACCCTCACTTCTTCTG
PPP1R14BP3 (chr4:140036046-140036095) (plus strand)
Match
SNP
GTCCTGTGGGAGCCACCGTTCTCCTGGGTCGGGGACCGTCACTTCTTCTG
PPP1R14B (chr11:64012188-64012237) (plus strand)
R1.26307012: GTCCTGTGGGAGCCACCGTTCTCCTGGGTCGGGGACCCTCACTTCTTCTG
R2.26307012: TGCTGGTTGACTGTTACAAACCCACAGAGGCCTTCATTTCTGGCCTGCTG
Soft clipping
CCAGAAATGAAGGCCTCTGTGGGTTTGTAACAGTCAACCAGCAGCTCCTT
PPP1R14BP3 (chr4:140036139-140036188) (minus strand)
Skipped
CAGCAGGCCAGAAATGAAGGCCTGGATGGGGTGGGAGGGTAAACATTAAG
PPP1R14B (chr11:64012274-64012323) (minus strand)

Additional Fig. S2. Comparisons of m^{6}A between pseudogenes and their cognate protein-coding genes in GM12878 and H1 cell line. **a** The GO enrichment of the cognate protein-coding genes of pseudogenes with valid m^{6}A levels in GM12878 cell line. **b** GO enrichment plot showing the GO enrichment of the cognate protein-coding genes of pseudogenes with valid m^{6}A levels in H1 cell line. **c** Box plot comparing the m^{6}A levels between pseudogenes and their cognate protein-coding genes for multi-exon pseudogenes and intronless (single-exon) pseudogenes respectively in GM12878 cell line. **d** Scatter plot comparing the m^{6}A levels between pseudogenes and their cognate protein-coding genes for multi-exon pseudogenes and intronless (single-exon) pseudogenes respectively in GM12878 cell line. **e** Box plot comparing the m^{6}A levels between pseudogenes and their cognate protein-coding genes for multi-exon pseudogenes and intronless (single-exon) pseudogenes respectively in H1 cell line. **f** Scatter plot comparing the m^{6}A levels between pseudogenes and their cognate protein-coding genes for multi-exon pseudogenes and intronless (single-exon) pseudogenes respectively in H1 cell line.

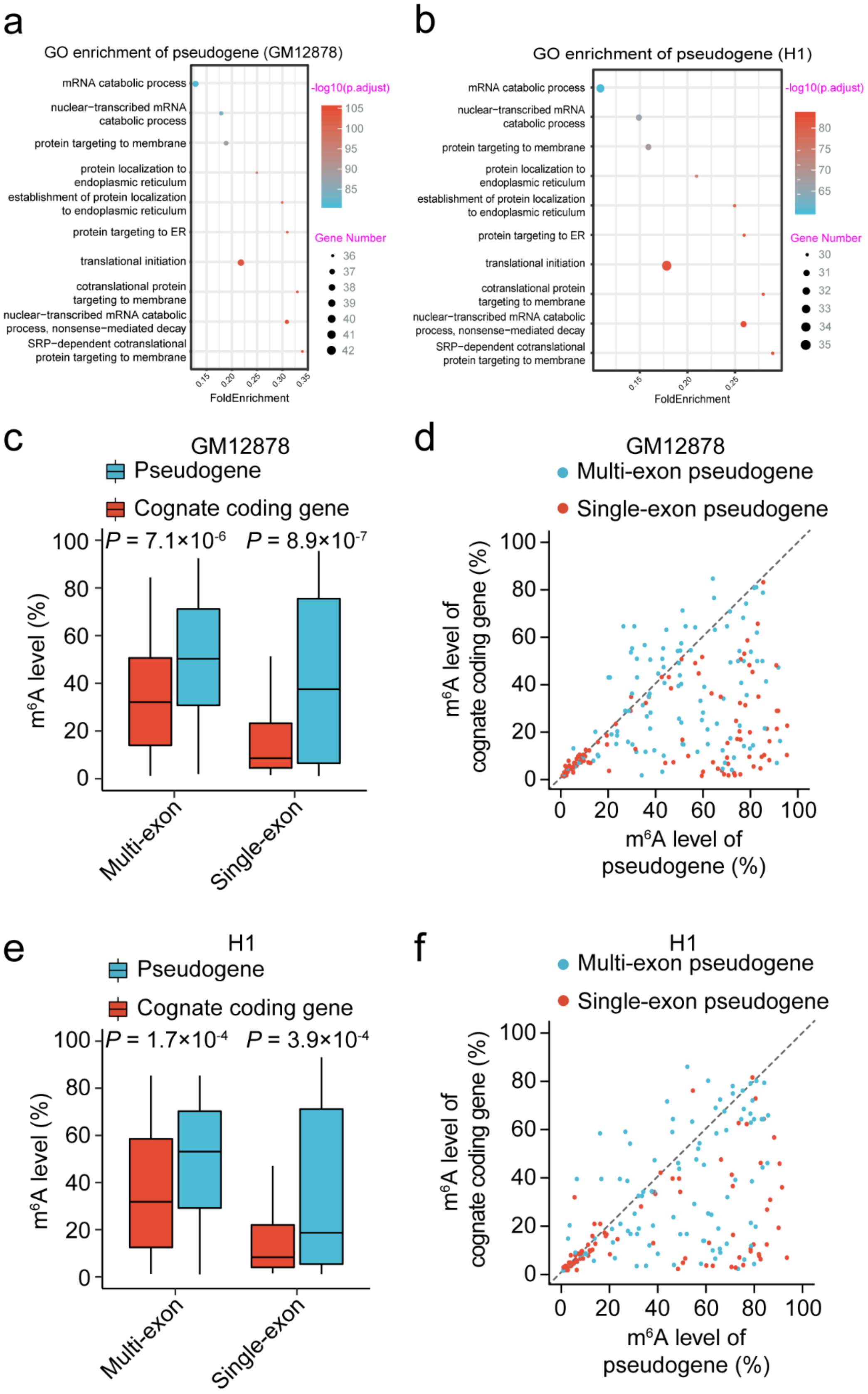
a
GO enrichment of pseudogene (GM12878)
mRNA catabolic process
nuclear−transcribed mRNA catabolic process
protein targeting to membrane
protein localization to endoplasmic reticulum
establishment of protein localization to endoplasmic reticulum
protein targeting to ER
translational initiation
cotranslational protein targeting to membrane
nuclear−transcribed mRNA catabolic process, nonsense−mediated decay
SRP−dependent cotranslational protein targeting to membrane
-log10(p.adjust)
Gene Number
FoldEnrichment
b
GO enrichment of pseudogene (H1)
c
GM12878
Pseudogene
Cognate coding gene
P = 7.1×10-6
P = 8.9×10-7
m6A level (%)
Multi-exon
Single-exon
d
Multi-exon pseudogene
Single-exon pseudogene
m6A level of cognate coding gene (%)
m6A level of pseudogene (%)
e
H1
P = 1.7×10-4
P = 3.9×10-4
f

Additional Fig. S3. Comparisons of m^{6}A between pseudogenes and their cognate protein-coding genes in H1 cell line. a Box plot comparing the m^{6}A levels among different biotypes. **b** Box plot comparing the m^{6}A levels between pseudogenes and their cognate protein-coding genes for processed pseudogenes and unprocessed pseudogenes respectively. **c** Scatter plot comparing the m^{6}A levels between pseudogenes and their cognate protein-coding genes for processed pseudogenes and unprocessed pseudogenes respectively. **d** UCSC genome browser tracks of m^{6}A-seq and m^{6}A-LAIC-seq data showing representative examples of pseudogenes and their cognate protein-coding genes. Read-coverage tracks of input, m^{6}A-negative, and m^{6}A-positive fractions of m^{6}A-LAIC-seq shown along with overlay tracks of m^{6}A-seq (red for RIP and cyan for input; predicted m^{6}A sites in m^{6}A peaks are indicated by arrows). Read coverage (y-axis) of m^{6}A negative and m^{6}A positive are normalized as previously described to reflects the calculated m^{6}A levels (i.e., equal signals in m^{6}A positive (eluate) versus m^{6}A negative (supernatant) indicate m^{6}A levels of 50%), while input and m^{6}A-seq tracks are shown for optimal viewing. **e** Plot of sequence alignment showing the pseudogenes-specific m^{6}A motifs in the pseudogenes *DSTNP2* and *NAP1L4P1*.

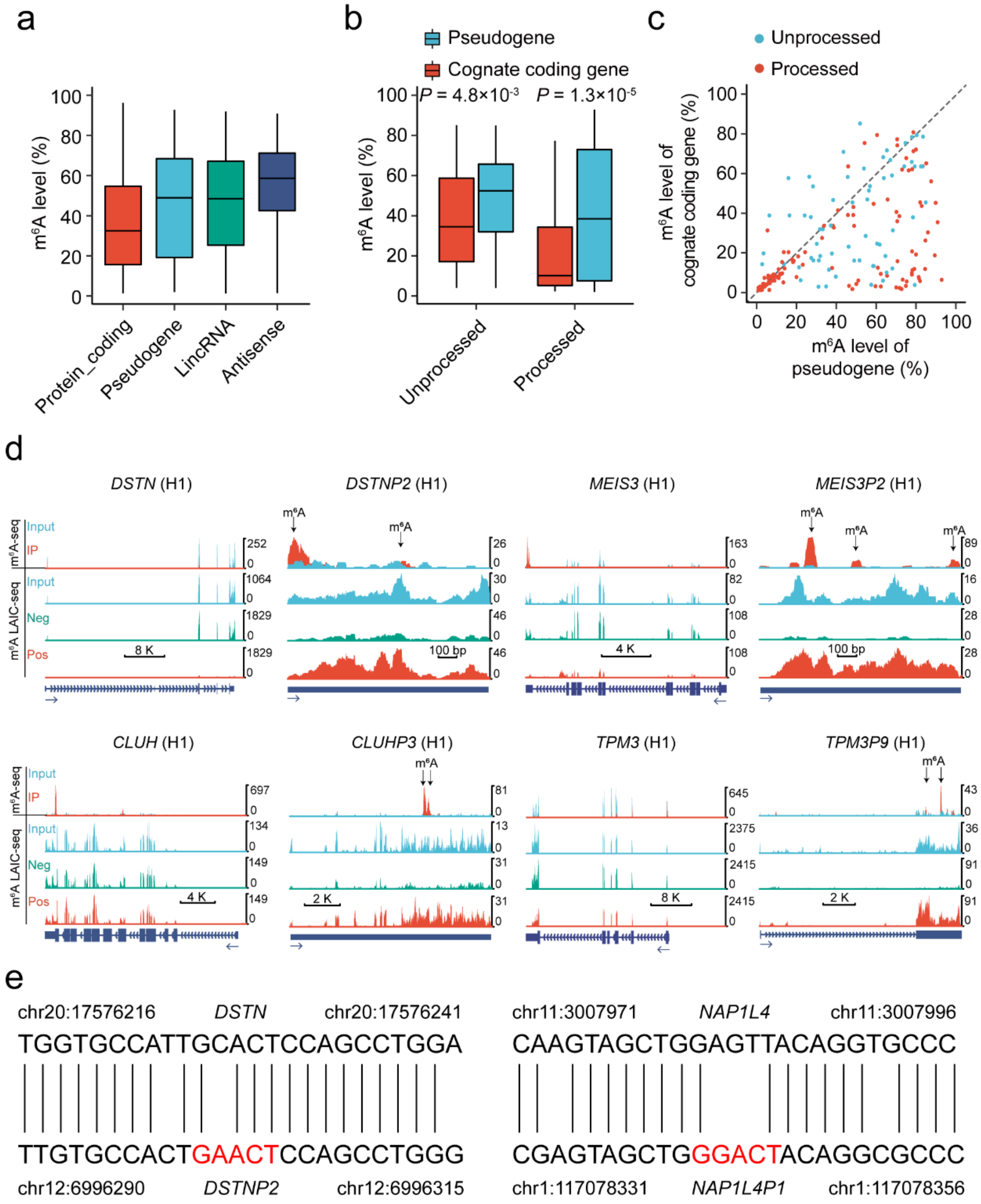
a
m6A level (%)
Protein_coding
Pseudogene
LincRNA
Antisense
b
Pseudogene
Cognate coding gene
P = 4.8×10-3
P = 1.3×10-5
m6A level (%)
Unprocessed
Processed
c
Unprocessed
Processed
m6A level of cognate coding gene (%)
m6A level of pseudogene (%)
d
DSTN (H1)
DSTNP2 (H1)
MEIS3 (H1)
MEIS3P2 (H1)
CLUH (H1)
CLUHP3 (H1)
TPM3 (H1)
TPM3P9 (H1)
m6A-seq
m6A LAIC-seq
Input
IP
Neg
Pos
m6A
e
chr20:17576216 DSTN chr20:17576241
TGGTGCCATTGCACTCCAGCCTGGA
TTGTGCCACTGAACTCCAGCCTGGG
chr12:6996290 DSTNP2 chr12:6996315
chr11:3007971 NAP1L4 chr11:3007996
CAAGTAGCTGGAGTTACAGGTGCCC
CGAGTAGCTGGGACTACAGGCGCCC
chr1:117078331 NAP1L4P1 chr1:117078356

Additional Fig. S4. Correlation of the m^{6}A of processed pseudogenes in GM12878 cells with the gene features of their cognate protein-coding genes. a-d Box plots comparing the m^{6}A levels among the processed pseudogenes with different ranges of 5'UTR length **a**, CDS length **b**, 3'UTR length **c**, and GC content **d** of their cognate coding genes.

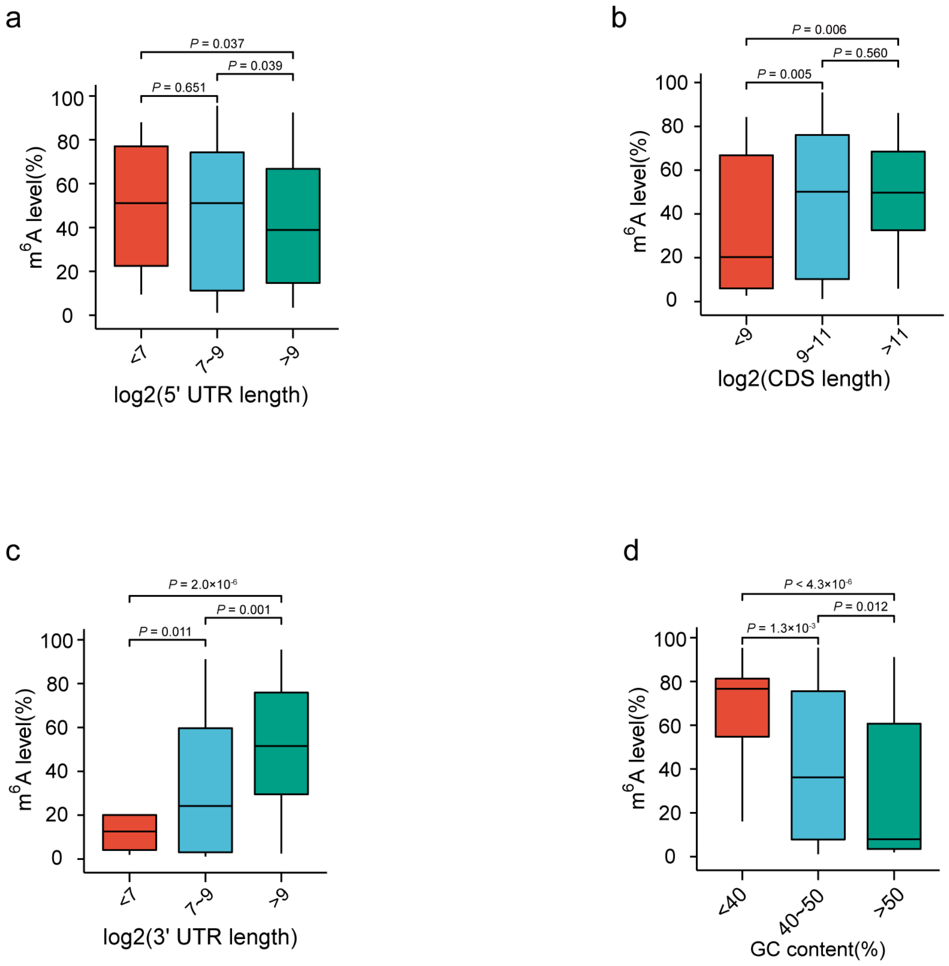

Additional Fig. S5. Positive natural selection of the m^{6}A on pseudogenes in H1 cell line. a The distributions of the ages of m^{6}A sites (left) and control non-m^{6}A DRACH sites (right). The age of each site was based on the most distantly related species in which the site was conserved. **b** Box plot comparing the peak intensities between m^{6}A sites with high (top 25%, strong constraint) and low (bottom 25%, weak constraint) rejected substitution scores. **c** Barplot showing the percentages of DRACH motifs within m^{6}A peaks of H1 cells for DRACH motif of different ages. **d** Box plot comparing the peak intensities of m^{6}A sites with different ages of pseudogenes. **e, f** Scatter plots showing the correlation of the sequence divergences between pseudogenes and their cognate protein-coding genes with the m^{6}A levels of pseudogenes for processed **e** and unprocessed **f** pseudogenes respectively. **g, h** Box plot comparing m^{6}A level of pseudogenes with different ranges of dN/dS ratios of their cognate coding genes for processed **g** and unprocessed **h** pseudogenes respectively. **i-l** Box plots comparing the m^{6}A levels among the processed pseudogenes with different ranges of 5'UTR length **i**, CDS length **j**, 3'UTR length **k**, and GC content **l** of their cognate coding genes.

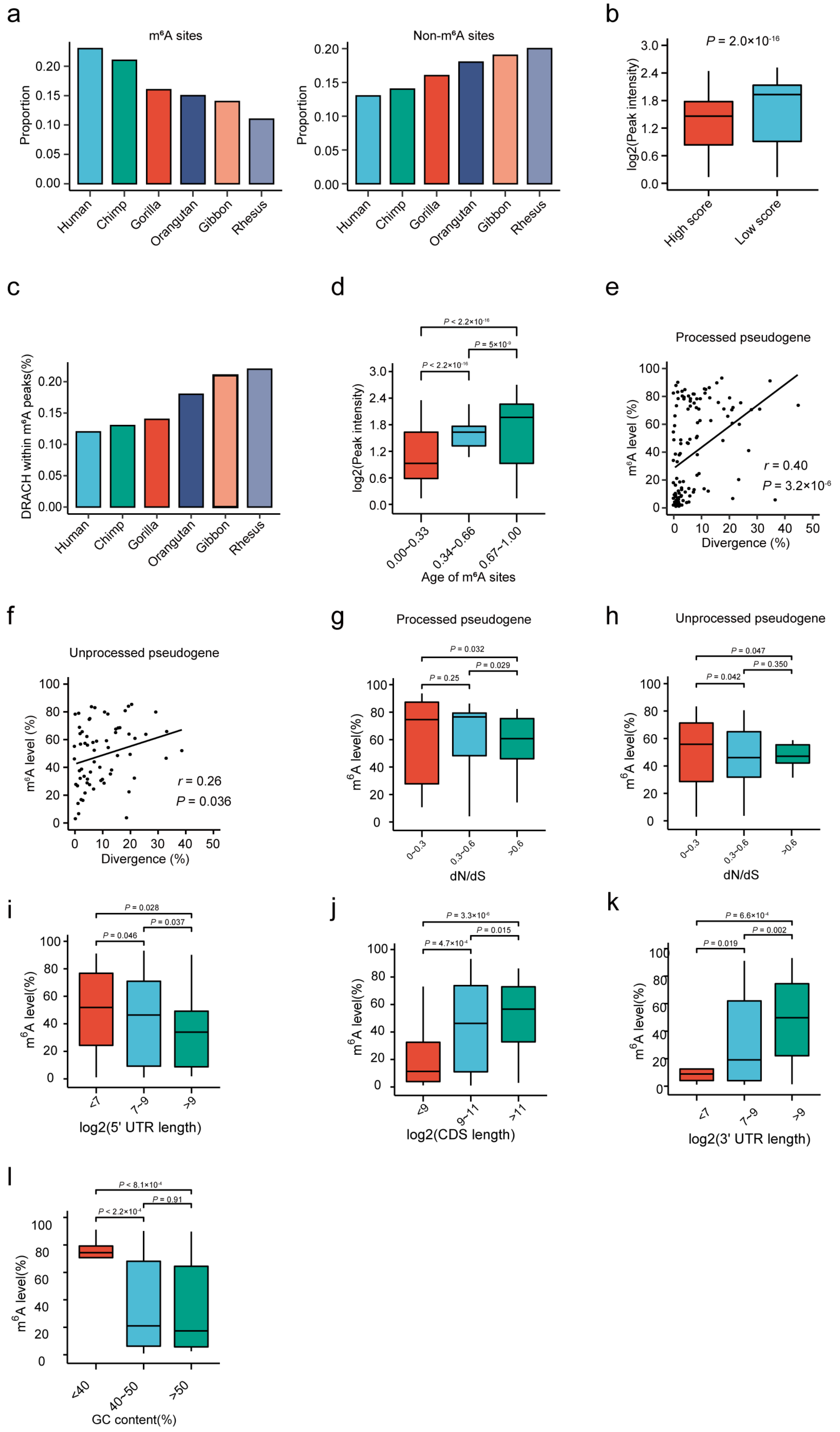

a
m6A sites
Non-m6A sites
Proportion
Human
Chimp
Gorilla
Orangutan
Gibbon
Rhesus
b
P = 2.0×10-16
log2(Peak intensity)
High score
Low score
c
DRACH within m6A peaks(%)
d
P < 2.2×10-16
P = 5×10-9
log2(Peak intensity)
0.00~0.33
0.34~0.66
0.67~1.00
Age of m6A sites
e
Processed pseudogene
m6A level (%)
r = 0.40
P = 3.2×10-6
Divergence (%)
f
Unprocessed pseudogene
r = 0.26
P = 0.036
g
Processed pseudogene
P = 0.032
P = 0.029
P = 0.25
m6A level(%)
dN/dS
h
Unprocessed pseudogene
P = 0.047
P = 0.350
P = 0.042
i
P = 0.028
P = 0.037
P = 0.046
log2(5' UTR length)
j
P = 3.3×10-6
P = 0.015
P = 4.7×10-4
log2(CDS length)
k
P = 6.6×10-4
P = 0.002
P = 0.019
log2(3' UTR length)
l
P < 8.1×10-4
P = 0.91
P < 2.2×10-4
GC content(%)

Additional Fig. S6. m⁶A facilitates the cytosolic degradation of cognate protein-coding genes of processed pseudogenes in GM12878 cell line. a, b Plot of cumulative fraction of expression **a** and nuclear index **b** for processed pseudogene with high (m6A level ≥ 0.6), medium (0.6 > m6A level ≥ 0.3), and low (m6A level < 0.3) m⁶A levels respectively in control cells. Two-tailed Wilcoxon *P* values are indicated at the bottom right corner. **c** Plot of cumulative fraction of expression of cognate protein-coding genes for unprocessed pseudogenes with high (m⁶A level ≥ 0.6), medium (0.6 > m⁶A level ≥ 0.3), and low (m⁶A level < 0.3) m⁶A levels respectively. Two-tailed Wilcoxon *P* values are indicated at the top left corner and bottom right corner, respectively. **d, e** Plot of cumulative fraction of nuclear indexes (ratio of expression between nucleus and cytosol) of cognate protein-coding genes for processed **d** and unprocessed **e** pseudogene with high, medium, and low m⁶A levels respectively. Two-tailed Wilcoxon *P* values are indicated at the bottom right corner.

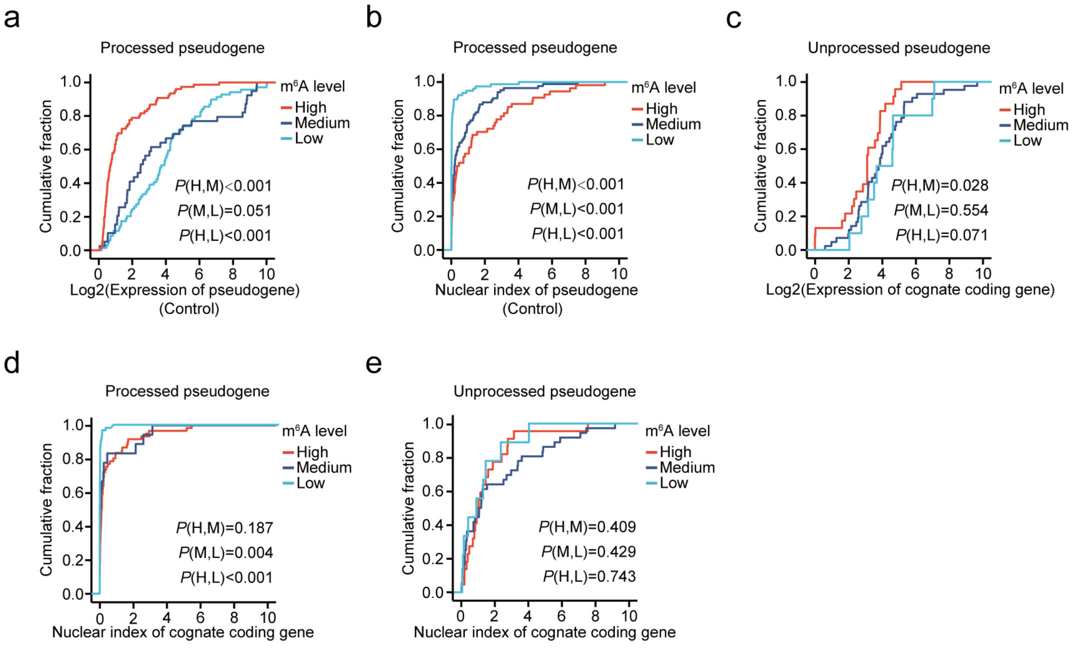

Additional Fig. S7. m^{6}A facilitates the cytosolic degradation of processed pseudogenes in H1 cell line. a, b Plot of cumulative fraction of expression for processed **a** and unprocessed **b** pseudogene with high (m^{6}A level ≥ 0.6), medium (0.6 > m^{6}A level ≥ 0.3), and low (m^{6}A level < 0.3) m^{6}A levels respectively. Two-tailed Wilcoxon *P* values are indicated at the bottom right corner. **c, d** Plot of cumulative fraction of nuclear indexes (ratio of expression between nucleus and cytosol) for processed **c** and unprocessed **d** pseudogene with high, medium, and low m^{6}A levels respectively. Two-tailed Wilcoxon *P* values are indicated at the bottom right corner. **e, f** Plot of cumulative fraction of cytosolic **e** and nuclear **f** gene expression for processed pseudogene with high, medium, and low m^{6}A levels respectively. Two-tailed Wilcoxon *P* values are indicated at the bottom right corner.

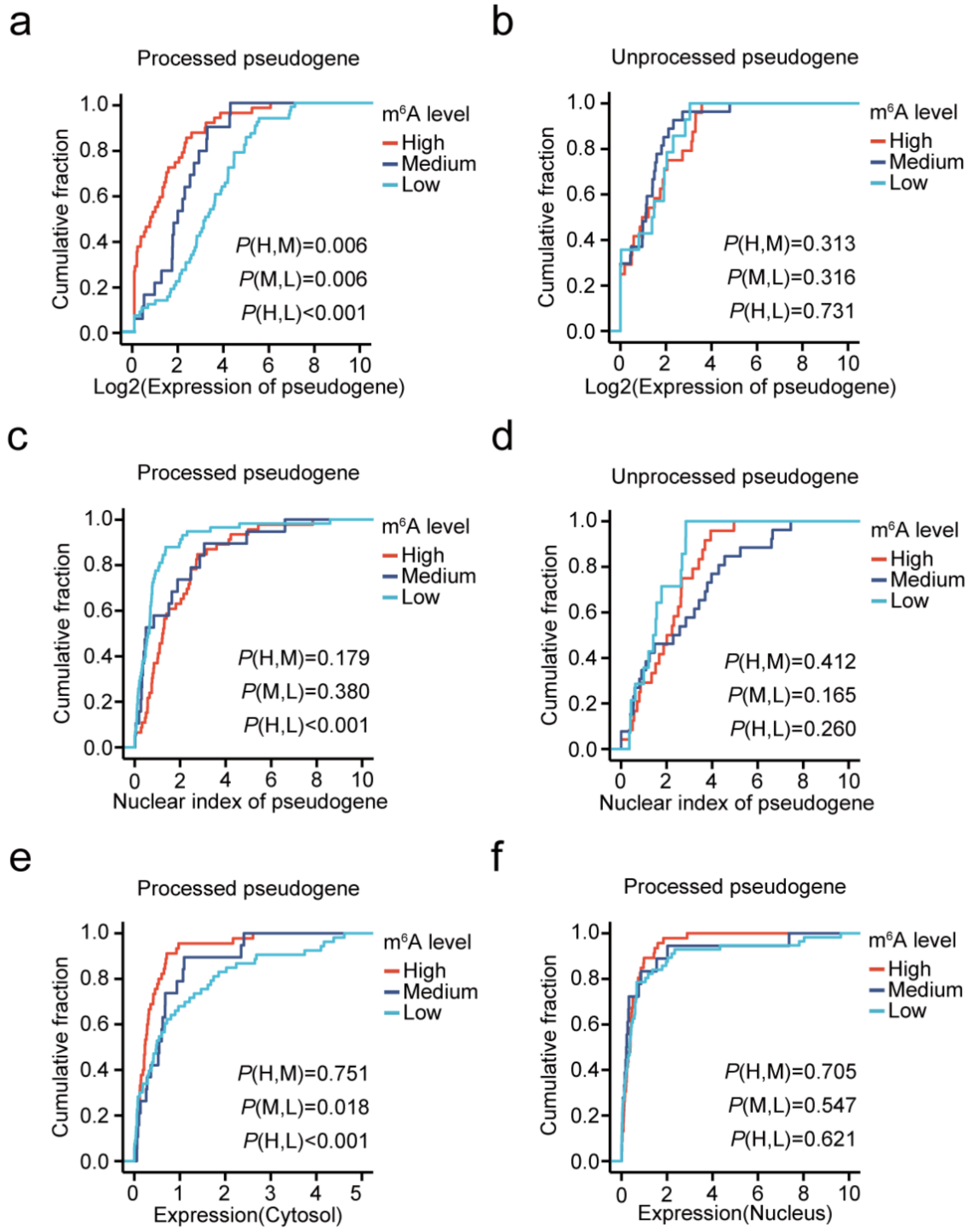

Additional Fig. S8. m^{6}A facilitates the cytosolic degradation of cognate protein-coding genes of processed pseudogenes in H1 cell line. a, b Plot of cumulative fraction of expression of cognate protein-coding genes for processed **a** and unprocessed **b** pseudogene with high (m^{6}A level ≥ 0.6), medium (0.6 > m^{6}A level ≥ 0.3), and low (m^{6}A level < 0.3) m^{6}A levels respectively. Two-tailed Wilcoxon *P* values are indicated at the top left corner and bottom right corner, respectively. **c, d** Plot of cumulative fraction of nuclear indexes (ratio of expression between nucleus and cytosol) of cognate protein-coding genes for processed **c** and unprocessed **d** pseudogene with high, medium, and low m^{6}A levels respectively. Two-tailed Wilcoxon *P* values are indicated at the bottom right corner.

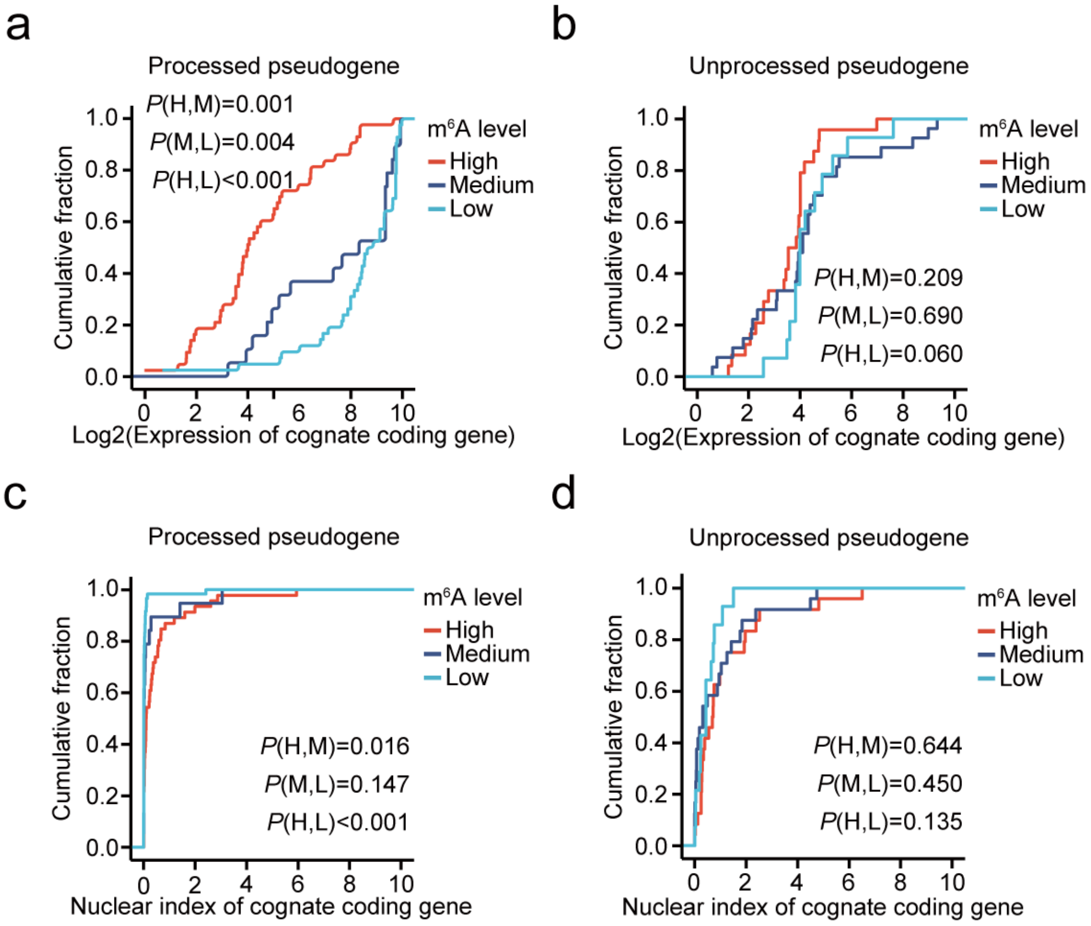

Additional Fig. S9 The gene regulatory network and primer design principles of processed pseudogenes and cognate protein-coding genes. a The processed pseudogene *DSTNP1*-miRNA-cognate protein-coding gene *DSTN* regulatory network. Pink round rectangles represented pseudogene *DSTN*, which is located at the left of the networks. Tomato ellipses represented binding miRNAs, which are located at the center of the networks. And blue round rectangles stand for cognate protein-coding gene *DSTN*, which is located at the right of the networks. **b** No mutations were shown in miR362-5p and miR3681-5p binding region on wild-type and m^{6}A site-mutated *DSTNP2* and *NAP1L4P2*, respectively. **c** The qPCR primer designs for *DSTN*, *DSTNP2*, *NAP1L4*, and *NAP1L4P2*, respectively.

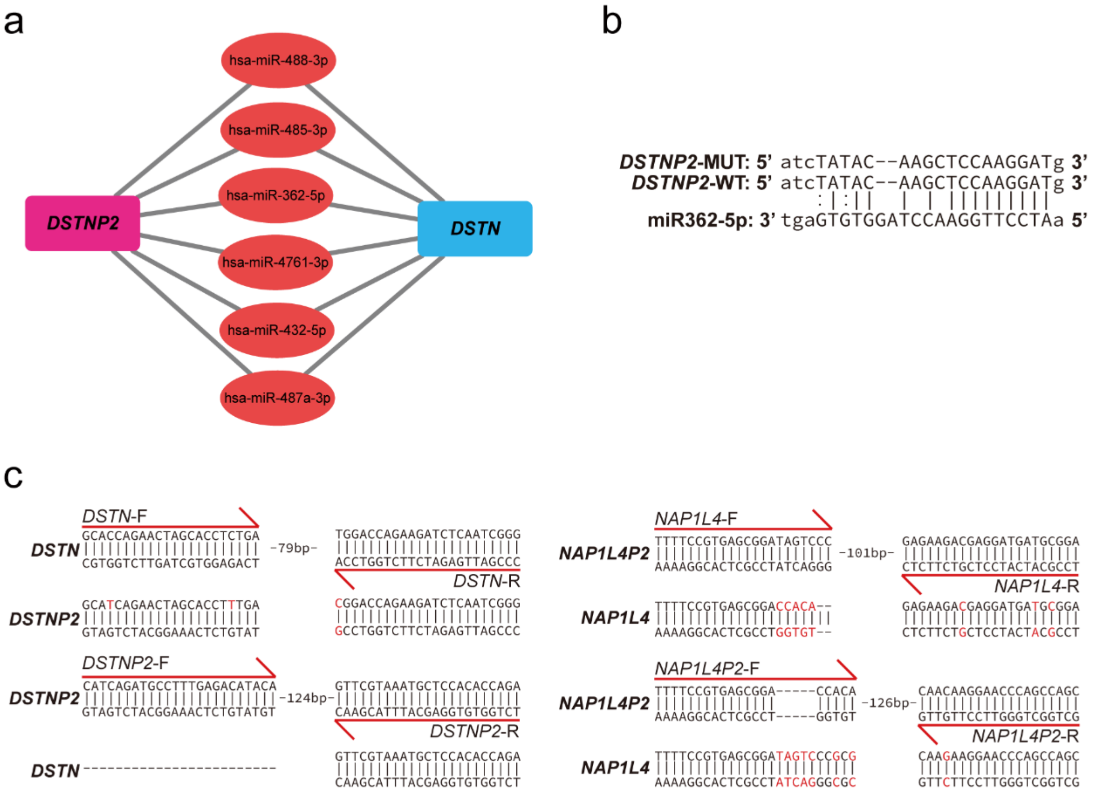

Additional Fig. S10. The uncropped western blot . a The uncropped western blot comparing the protein levels of *DSTN* in control and *DSTNP2* knockdown cells. **b** The uncropped western blot comparing the protein levels of DSTN upon overexpression of wild type *DSTNP2* versus m^{6}A site-mutated *DSTNP2*. **c** The uncropped western blot showing the protein level of *DSTN* upon overexpression of wild type *DSTNP2* versus m^{6}A site-mutated *DSTNP2* after adding control inhibitor and miR362-5p inhibitor respectively.

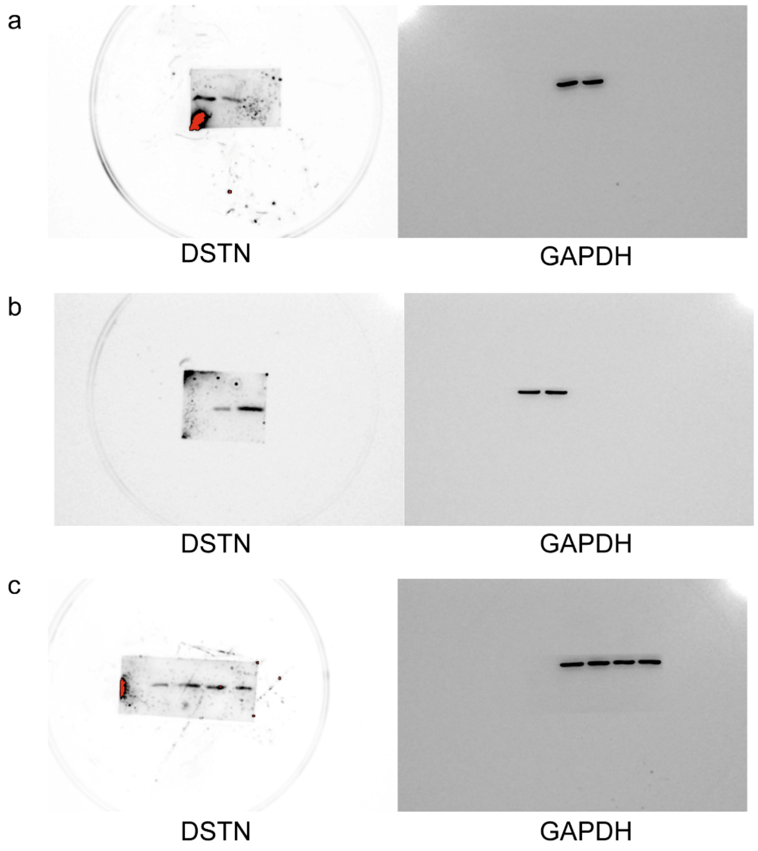

Additional Fig. S11. Experimental validation of m⁶A on processed pseudogene *NAP1L4P1* affects the expression of *NAP1L4* in GM12878 cell line. a The relative expression of *DSTNP2* and *NAP1L4P1* upon vector, overexpression wild type pseudogenes and overexpression m⁶A site-mutated pseudogenes, respectively. **b** The relative expression of *NAP1L4* upon overexpression empty vector and wild type *NAP1L4P1* respectively. Error bars represent standard errors. *** P<0.001 (two-tailed t test). **c** Bar plot comparing the relative expression of total, cytoplasmic and nuclear *NAP1L4* respectively upon overexpression of wild type versus m⁶A sites-mutated *NAP1L4P1*. **d, e** The relative expression of *NAP1L4P1* **d** and *NAP1L4* **e** upon overexpression of wild type *NAP1L4P1* versus m⁶A site-mutated *NAP1L4P1* at different time points after transcription inhibition. Error bars represent standard errors. ** P<0.01; *** P<0.001 (two-tailed t test).

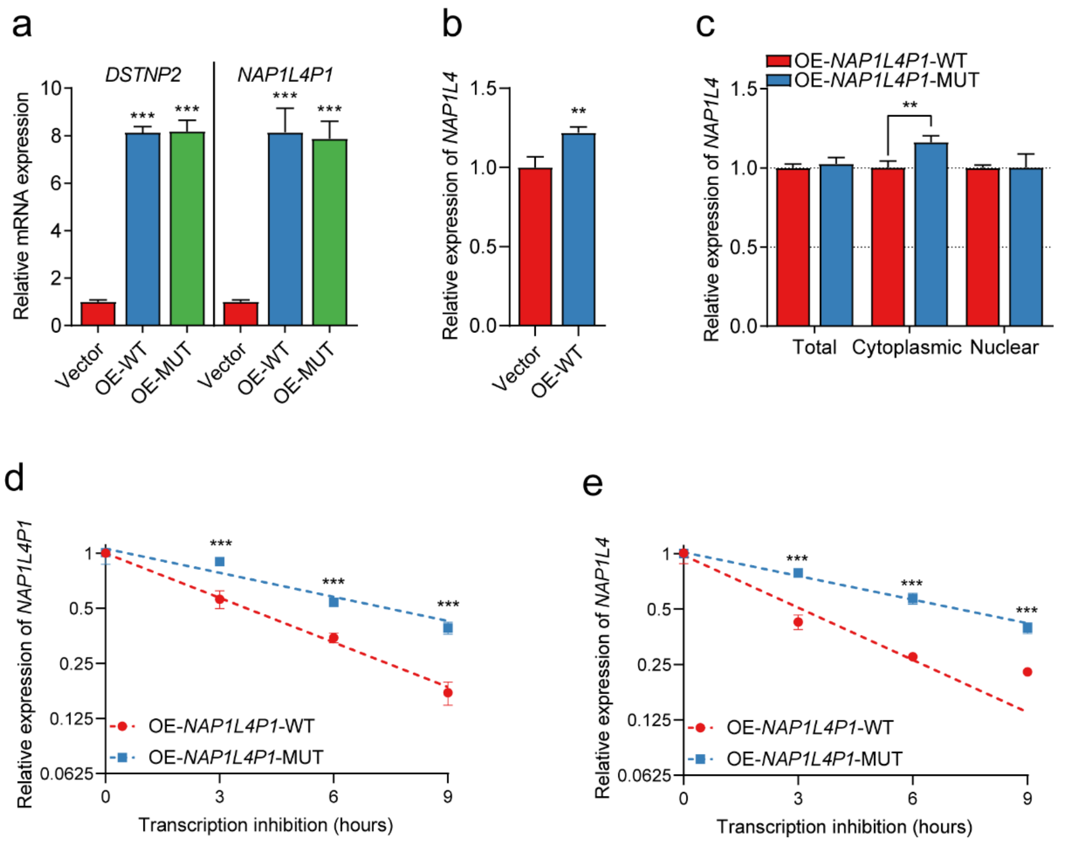

Additional Fig. S12. Correlation between m^{6}A levels and exon numbers of coding genes. a Boxplot comparing the m^{6}A levels between spliced coding genes and intronless coding genes in GM12878 cells. **b** Boxplot comparing the m^{6}A levels of coding genes with different number of exons in GM12878 cells. **c** Boxplot comparing the m^{6}A levels between spliced coding genes and intronless coding genes in H1 cells. **d** Boxplot comparing the m^{6}A levels of coding genes with different number of exons in H1 cells.

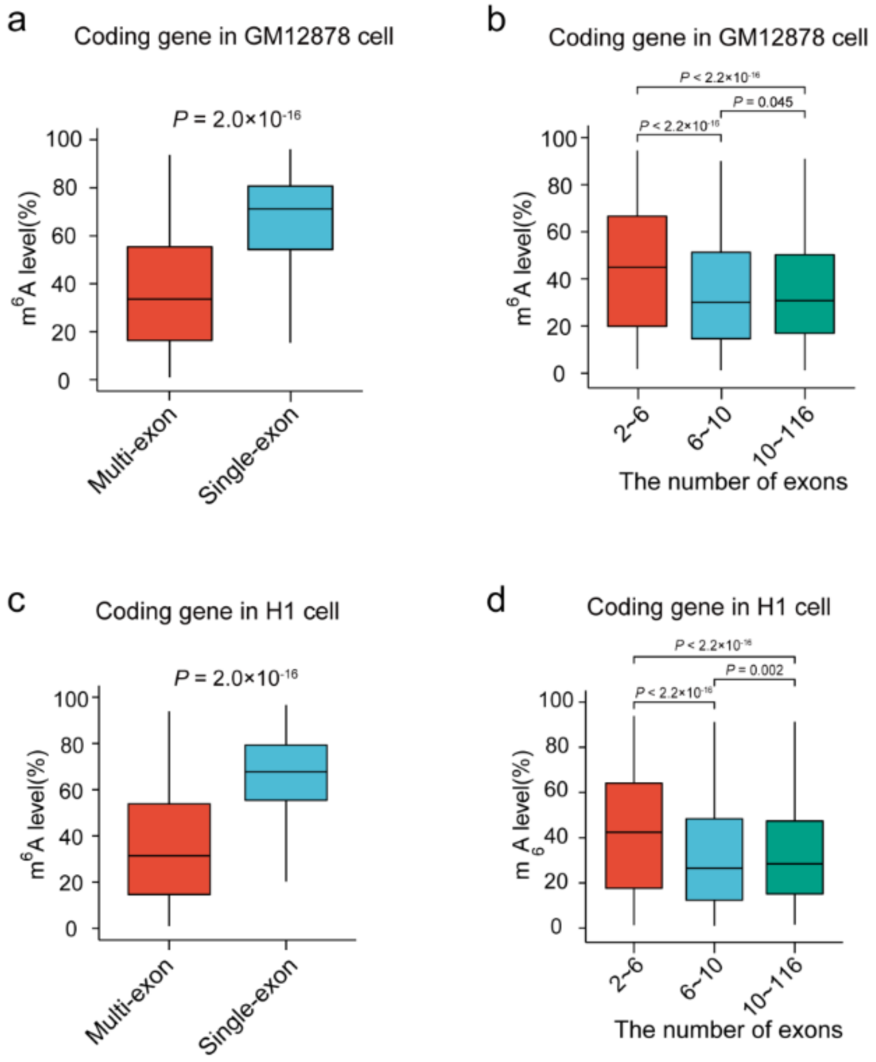

Supplement: Supplementary file 1 — Additional file 1: Figure S1. The optimization of conventional HISAT2 mapping procedure. Figure S2. Comparisons of m6A between pseudogenes and their cognate protein-coding genes in GM12878 and H1 cell line. Figure S3. Comparisons of m6A between pseudogenes and their cognate protein-coding genes in H1 cell line. Figure S4. Comparisons of pseudogenes m6A among 5′UTRs, CDSs, 3′UTRs and GC contents in their cognate protein-coding genes in GM12878 cell line. Figure S5. Positive natural selection of the m6A on pseudogenes in H1 cell line. Figure S6. m6A facilitates the cytosolic degradation of cognate protein-coding genes of processed pseudogenes in GM12878 cell line. Figure S7. m6A facilitates the cytosolic degradation of processed pseudogenes in H1 cell line. Figure S8. m6A facilitates the cytosolic degradation of cognate protein-coding genes of processed pseudogenes in H1 cell line. Figure S9. The gene regulatory network and primer design principles of processed pseudogenes and cognate protein-coding genes. Figure S10. The uncropped bots of western blots in Fig. 5. Figure S11. Experimental validation of m6A on processed pseudogene NAP1L4P1 affects the expression of NAP1L4 in GM12878 cell line. Figure S12. Correlation between m6A levels and exon numbers of coding genes. [file 13059_2021_2402_MOESM1_ESM.pdf]
