# Supplementary material for: Positive natural selection of N6-methyladenosine on the RNAs of processed pseudogenes
Source: Genome Biol. 2021 Jun 13;22:180. doi: 10.1186/s13059-021-02402-2 (PMC8201931; doi:10.1186/s13059-021-02402-2)
Supplement: Supplementary file 3 — Additional file 3. Review history. [file 13059_2021_2402_MOESM3_ESM.docx]

**1^st^ round**

**Reviewer 1**

In this paper, Tan et al., found that processed pseudogenes tend to contain more m6A sites compared with the corresponding cognate protein-coding gene. Then, the authors showed that m6A facilitates the cytosolic degradation of processed pseudogenes. In addition, they claimed that m6A of processed pseudogenes disrupts a crosstalk with their cognate protein-coding genes. Overall, although the authors found a very interesting phenomenon on the preferential m6A in processed pseudogenes, their major conclusions require additional supporting evidence.

Specific comments:

1. Abstract: "Nonsense Mediated Decay (NMD) is an important splicing dependent process for RNA surveillance in mammals". This sentence should be changed to "Nonsense Mediated mRNA Decay (NMD) is an important splicing dependent process for mRNA surveillance in mammals.

2. line 59: "Splicing independent NMD like in yeast has not been found in mammals, and it is widely known that intronless genes are insensitive to NMD". This sentence is very misleading, because one of NMD features includes a long 3'UTR. Therefore, some mRNAs are targeted for NMD, although they do not contain an intron.

3. Figure 1: All data clearly support the authors' claim for a preferential m6A in processed pseudogenes. If so, why do the processed pseudogenes exhibit more m6A level, compared to cognate protein-coding genes? How are the processed pseudogenes selectively m6A methylated?

4. Figure 2: DRACH is consensus motif for m6A modification. However, I doubt whether all DRACH motifs are m6A methylated.

5. Figure 3: There are several ways to inhibit cytosolic degradation of m6A RNAs. What happens to pseudogenes under conditions lacking m6A methylase.

6. Figure 5: It is expected that downregulation of endogenous DSTNP2 by siRNA may increase the level of DSTN mRNA. I think that this additional experiment could provide strong evidence supporting the authors' claim. In addition, in panel C and F, the effect of overexpressed Mut is very weak.

===

Please consider the following questions:

- Are the methods appropriate to the aims of the study, are they well described, and are necessary controls included? If not, please specify what is required.

- Are the conclusions adequately supported by the data shown? If not, please explain

- Are sufficient details provided to allow replication and comparison with related analyses that may have been performed? If not, please specify what is required.

- Does the work represent a significant advance over previously published studies?

- Is the paper of broad interest to others in the field, or of outstanding interest to a broad audience of biologists?

**Reviewer 2**

In this manuscript, the authors provide a series of evidence supporting a novel role of the transcript modification m6A of processed pseudogenes. I believe it will be interesting to the readers of Genome Biology. The manuscript is well written. I have only a few comments for the authors to consider in their revision of this manuscript.

The m6A is a modification on the transcripts rather than on the genes or pseudogenes. If just reading the abstract, readers without prior knowledge on the subject would have an impression of modifications on the genes, i.e. another type of DNA methylation. Although after reading the main text, readers would know where the modifications are, there are many sentences misleading, e. g., "suggesting that methylation of pseudogenes may interplay with their cognate protein-coding genes." The interplay happens between their RNAs, rather than the genes directly. With the advance of 3D structural analysis of the sequence interactions in nucleus, we may see interplays between genes. I strongly suggest the authors to revise their wordings.

The authors claimed that the m6A motifs on pseudogenes are under positive natural selection, based on several pieces of evidence. I agree that the adaptive selection or called positive natural selection is the most convincing conclusion. However, I do not think adaptive selection is the only hypothesis. Strand asymmetric mutations could also explain the different frequency of appearance of the m6A motifs.[1, 2] between RNAs originated from different strands . I hope the authors could tone down their conclusion using phrases like, evidence supporting that m6A motifs on pseudogenes are under positive natural selection.

While reading pages 8-9, I hope to see whether the m6A levels of processed pseudogenes are correlated with some genomic features of their cognate protein-coding genes. And in page 11, I am very happy to see that it is negatively correlated with the expression level of the cognate protein-coding genes. Please check whether the m6A levels of processed pseudogenes are correlated with the essentiality or level of conservation of their cognate protein-coding genes, whether important genes are more likely regulated by the modification of their pseudogene transcripts.

In the last sentence of the Discussion section, the authors said, "it is possible that m6A mediated RNA degradation also plays important roles for RNA surveillance of intronless protein-coding genes." Why not survey the level of m6A in the transcripts of intronless protein-coding genes. Although just the level of m6A is not enough to test the hypothesis, the data itself is interesting and relevant to this study. It is also interesting to see whether the level of transcript m6A are correlated with the number of introns, or length of the protein-coding genes.

Are there any specific motifs associated with the m6A modification? If previous studies have done such work, please cited them.

1. Francino MP, Ochman H. (1997). Strand asymmetries in DNA evolution. Trends Genet. 13: 240-245.

2. Haradhvala NJ, Polak P, Stojanov P, et al. (2016). Mutational Strand Asymmetries in Cancer Genomes Reveal Mechanisms of DNA Damage and Repair. Cell 164: 538-549.

**Reviewer 3**

Authors study N6-methyladenosine (m6A) RNA modification in pseudogenes. They use their previously published data of a human B-lymphoblastoid cell line and a human embryonic stem cell line. They observe that processed pseudogenes quickly evolved m6A RNA modifications as compared to their cognate coding gene. The m6A RNA motifs also increase peak intensity with motif age/conservation. They interpret this data and the lower level of expression of highly m6A modified pseudogenes as adaptations given the role of this RNA modification on transcript degradation. Authors experimentally validated some of their results. All of this makes this work interesting to a broad audience. I have several question that I would like authors to address to understand how well supported their inferences are.

Major comments:

1. Why do authors think that there are only 223 pseudogenes with reliable m6A levels out of 12,143 in this study? Wouldn't we expect more, if this trait is under selection?

2. If I understand correctly, authors improve their mapping of the reads to study the m6A levels compared to previous study. They consider the new approach more reliable. Is there a way to confirm that this is the case?

3. In figure 1b, if seems that what is different are cognate coding genes of processed pseudogenes. These cognate coding genes are the ones that show low m6A level percent. Are these cognate coding genes different in some way that explains their low m6A level?

4. In figure 2 g, it seems that processed pseudogenes evolve high methylation level very quickly. Is this the case?

5. In the past, even processed copies of genes that do not have disablements have been called processed pseudogenes. So, authors should clarify what kinds of disablements the pseudogenes considered in this work have.

Other comments:

1. Line 77-80. Processed and unprocessed pseudogenes are duplicates. Processed pseudogenes are duplicates that were produced through an mRNA intermediate and unprocessed pseudogenes are duplicates that were produced through an DNA intermediate.

2. Line 82. I would say "…lack of intronS."

3. Line 144. Remove et al.

4. Line 174. I would say "The m6A motifs on pseudogenes EVOLVED under positive natural selection."

5. Line 314. I think authors mean "neutral"

**Authors’ response**

Reviewer #1:

In this paper, Tan et al., found that processed pseudogenes tend to contain more m6A sites compared with the corresponding cognate protein-coding gene. Then, the authors showed that m6A facilitates the cytosolic degradation of processed pseudogenes. In addition, they claimed that m6A of processed pseudogenes disrupts a crosstalk with their cognate protein-coding genes. Overall, although the authors found a very interesting phenomenon on the preferential m6A in processed pseudogenes, their major conclusions require additional supporting evidence.

Specific comments:

1. Abstract: "Nonsense Mediated Decay (NMD) is an important splicing dependent process for RNA surveillance in mammals". This sentence should be changed to

"Nonsense Mediated mRNA Decay (NMD) is an important splicing dependent process

for mRNA surveillance in mammals.

Author Response: Thanks for the reviewer’s comment! We have changed this sentence.

2. line 59: "Splicing independent NMD like in yeast has not been found in mammals, and it is widely known that intronless genes are insensitive to NMD". This sentence is very misleading, because one of NMD features includes a long 3'UTR. Therefore, some mRNAs are targeted for NMD, although they do not contain an intron.

Author Response: We thank the reviewer for pointing out this issue! We have briefly introduced the effects of long 3’UTR on NMD and changed the sentences as the following (page 3):

“*However, the canonical NMD becomes a splicing dependent process in mammals, it can be triggered as long as the stop codons are more than 50~55 bp upstream of the last exon-exon junction. Although NMD can also be triggered by very long 3’UTR (Cell.2010,143:379-89), intronless genes are more likely insensitive to NMD*.”

3. Figure 1: All data clearly support the authors' claim for a preferential m6A in processed pseudogenes. If so, why do the processed pseudogenes exhibit more m6A level, compared to cognate protein-coding genes? How are the processed pseudogenes selectively m6A methylated?

Author Response: Thanks for the reviewer’s comment! The elevated m6A levels of processed pseudogenes are driven by natural selection of the m6A motifs on the processed pseudogenes. Random mutations that generate m6A motifs on processed pseudogenes are more likely fixed in the evolution because they are adaptive, therefore, processed pseudogenes can accumulate more m6A motifs and become more and more highly methylated during the evolution. In contrast, the m6A motifs on the cognate coding gene tend to be conserved, therefore, random mutations that increase m6A may be deleterious because they may disrupt the homeostasis thus tend to be lost in the evolution. We have a paragraph in the DISCUSSION to address this question (page 16-17).

“*Here, we found a novel mechanism of RNA surveillance for processed pseudogenes via m6A RNA methylation. How does this mechanism evolve? In this study, we found significantly higher probability of obtaining novel m6A motifs on sense strand than antisense strand, strongly suggest that obtaining m6A sites on pseudogenes tend to be adaptive and accumulating in the evolution. In this way, the elevated m6A on processed pseudogenes are the results of positively selected mutations that produce novel m6A sites. Alternatively, we cannot rule out there is unknown specific mechanism, such as RBP mediated specific regulation of m6A (Nucleic Acids Res.2020,48:1715-29), to recognize processed pseudogenes and mark them for cytosolic degradation by m6A RNA modification. However, whether and how the cells mark processed pseudogenes is still unknown and require further investigation*.”

4. Figure 2: DRACH is consensus motif for m6A modification. However, I doubt whether all DRACH motifs are m6A methylated.

Author Response: Thanks for the reviewer’s comment! We have analyzed the proportions of DRACH motifs that within m6A peaks of GM12878 and H1 cells for the DRACH motifs originated at different phylogenic branches respectively, we found the older the motifs are, the higher proportions of them to be m6A modified (Fig. 2f). There are only 13% of human-specific DRACH motifs m6A modified in GM12878 cells. In contrast, 22% of human and rhesus shared DRACH are m6A modified in GM12878 cells. Similar result was also observed in H1 cells (Additional file 1: Fig. S5c). These results suggest that generating of DRACH motifs is the first step, it still takes time for the motifs to get m6A methylated in the evolution. In addition, although most of the DRACH motifs are not methylated strong enough to show detectable m6A peaks, they are quite possibly methylated at undetectable low level and gradually getting stronger in the evolution. We added the above sentences in the RESULTS (page 9):

5. Figure 3: There are several ways to inhibit cytosolic degradation of m6A RNAs. What happens to pseudogenes under conditions lacking m6A methylase.

Author Response: Thanks for the reviewer’s comment! We have sequenced the input, cytoplasmic, and nuclear RNAs of control and METTL3 knockdown GM12878 cells respectively. We found there are no significant difference of gene expression or nuclear index among the processed pseudogenes with high, median, and low m6A levels, suggesting that these differences are due to m6A. We have added the following sentences in the RESULTS (page 10-11).

“*To further confirm whether different expression of processed pseudogenes is due to m6A differences, we sequenced the input, cytoplasmic, and nuclear RNAs of control and METTL3 knockdown GM12878 cells respectively. Expression differences among the processed pseudogenes with different class of m6A levels were also observed in control cells (Additional file 1: Fig. S6a). However, the differences become not significant in METTL3 knockdown cells, indicating the expression differences of processed pseudogenes are due to m6A differences (Fig. 3c).*”

“*Similar results for processed pseudogenes were also observed in control GM12878 cells (Additional file 1: Fig. S6b), but the differences were not significant in METTL3 knockdown cells, indicating the nuclear index differences of processed pseudogenes are due to m6A differences (Fig. 3f).*”

6. Figure 5: It is expected that downregulation of endogenous DSTNP2 by siRNA may increase the level of DSTN mRNA. I think that this additional experiment could provide strong evidence supporting the authors' claim. In addition, in panel C and F, the effect of overexpressed Mut is very weak.

Author Response: Thanks for the reviewer’s comment! We guess the reviewer mean “It is expected that downregulation of endogenous DSTNP2 by siRNA may decrease the level of DSTN mRNA”, because DSTNP2 promotes the expression of DSTN by sponge miRNAs according to our proposed model. We have knocked down DSTNP2 using siRNA and found significantly decreased the gene expression and stability of DSTN, which is consistent with our previous finding that overexpression of DSTNP2 increases the expression and stability of DSTN RNAs (Fig. 5a, d).

In addition, although the effect of overexpressed Mut on steady state gene expression of DSTN is not strong, the effect on RNA stability is much stronger. Since cytoplasmic degradation directly reduced the RNAs ready for translation, we suspect it may have larger effect on protein level. First, we found knockdown DSTNP2 markedly decreased the protein level of DSTN (Fig. 5b). Comparing with overexpression of wild type DSTNP2, overexpression of DSTNP2 mutant resulted in markedly increased protein level of DSTN (Fig. 5g), and this effect was disrupted by inhibiting miR-362-5p (Figure 5l), consistent with our previous finding that the m6A of DSTNP2 decreases the RNA stability of DSTN partially through miR-362-5p. We have added the above results in the RESULTS section of the manuscript (page 13-14):

Reviewer #2:

In this manuscript, the authors provide a series of evidence supporting a novel role of the transcript modification m6A of processed pseudogenes. I believe it will be interesting to the readers of Genome Biology. The manuscript is well written. I have only a few comments for the authors to consider in their revision of this manuscript.

1.The m6A is a modification on the transcripts rather than on the genes or pseudogenes. If just reading the abstract, readers without prior knowledge on the subject would have an impression of modifications on the genes, i.e. another type of DNA methylation. Although after reading the main text, readers would know where the modifications are, there are many sentences misleading, e. g., "suggesting that methylation of pseudogenes may interplay with their cognate protein-coding genes."

The interplay happens between their RNAs, rather than the genes directly. With the advance of 3D structural analysis of the sequence interactions in nucleus, we may see interplays between genes. I strongly suggest the authors to revise their wordings.

Author Response: Thanks for the reviewer’s comment! We have revised the wording throughout the manuscript from title to avoid misleading readers.

2. The authors claimed that the m6A motifs on pseudogenes are under positive natural selection, based on several pieces of evidence. I agree that the adaptive selection or called positive natural selection is the most convincing conclusion. However, I do not think adaptive selection is the only hypothesis. Strand asymmetric mutations could also explain the different frequency of appearance of the m6A motifs.[1, 2] between RNAs originated from different strands . I hope the authors could tone down their conclusion using phrases like, evidence supporting that m6A motifs on pseudogenes are under positive natural selection.

Author Response: Thanks for the reviewer’s comment! We have toned down the conclusion throughout the manuscript as the reviewer advised.

3. While reading pages 8-9, I hope to see whether the m6A levels of processed pseudogenes are correlated with some genomic features of their cognate proteincoding genes. And in page 11, I am very happy to see that it is negatively correlated with the expression level of the cognate protein-coding genes. Please check whether the m6A levels of processed pseudogenes are correlated with the essentiality or level of conservation of their cognate protein-coding genes, whether important genes are more likely regulated by the modification of their pseudogene transcripts.

Author Response: Thanks for the reviewer’s comment! We have performed the analyses and added the following sentences in the RESULTS (page 10).

“*Interestingly, highly m6A methylated pseudogenes, especially processed pseudogenes, tend to have cognate protein-coding genes with lower dN/dS ratios, which represent the genes with higher essentiality and stronger functional constraint in the evolution, suggesting that important genes are more likely regulated by the modification of their processed pseudogene transcripts (Fig. 2j, k). On the other hand, we found the the highly m6A methylated processed pseudogenes tend to have cognate protein-coding genes with shorter 5’UTRs, longer CDSs and 3’UTRs, lower GC contents, suggesting that these gene features of cognate coding genes may also relate to the m6A evolution of processed pseudogenes (Additional file 1: Fig. S4a-d). Similar results were observed in H1 cells (Additional file 1: Fig. S5a-l)*.”

4. In the last sentence of the Discussion section, the authors said, "it is possible that m6A mediated RNA degradation also plays important roles for RNA surveillance of intronless protein-coding genes." Why not survey the level of m6A in the transcripts of intronless protein-coding genes. Although just the level of m6A is not enough to test the hypothesis, the data itself is interesting and relevant to this study. It is also interesting to see whether the level of transcript m6A are correlated with the number of introns, or length of the protein-coding genes.

Author Response: Thanks for the reviewer’s comment! Our previously published paper about m6A-LAIC-seq has reported that m6A levels are positively correlated with the 3’UTR length, which may reflect the relationship between m6A and alternative polyadenylation (Nature Methods. 2016,13(8):692-8). Here, we performed additional analyses and added the following sentences in the DISCUSSION (page 17-18).

“*we found the RNAs of protein-coding genes with single exons and limited number of exons also showed significantly higher m6A levels than genes with larger number of exons, suggesting that m6A mediated RNA degradation may also play roles for RNA surveillance of protein-coding genes with single or a few of exons (Additional file 1: Fig. S11a-d).*”

5. Are there any specific motifs associated with the m6A modification? If previous studies have done such work, please cited them.

Author Response: Thanks for the reviewer’s comment! m6A occurs at DRACH motifs on RNA. We have added the following sentence with citation in the BACKGROUND (page 4).

“*It is installed on DRACH motifs of RNAs (Nature Methods.2015,12:767-72.) by m6A methyltransferases complex with METTL3 as the catalytic subunit*.”

1.Francino MP, Ochman H. (1997). Strand asymmetries in DNA evolution. Trends Genet. 13: 240-245.

2.Haradhvala NJ, Polak P, Stojanov P, et al. (2016). Mutational Strand Asymmetries in Cancer Genomes Reveal Mechanisms of DNA Damage and Repair. Cell 164: 538- 549.

Reviewer #3:

Authors study N6-methyladenosine (m6A) RNA modification in pseudogenes. They use their previously published data of a human B-lymphoblastoid cell line and a human embryonic stem cell line. They observe that processed pseudogenes quickly evolved m6A RNA modifications as compared to their cognate coding gene. The m6A RNA motifs also increase peak intensity with motif age/conservation. They interpret this data and the lower level of expression of highly m6A modified pseudogenes as adaptations given the role of this RNA modification on transcript degradation. Authors experimentally validated some of their results. All of this makes this work interesting to a broad audience. I have several question that I would like authors to address to understand how well supported their inferences are.

Major comments:

1.Why do authors think that there are only 223 pseudogenes with reliable m6A levels out of 12,143 in this study? Wouldn't we expect more, if this trait is under selection?

Author Response: Thanks for the reviewer’s comment! The 12143 are the annotated pseudogenes. However, most of pseudogenes are not transcribed (RNA.2011,17(5): 792–8), and we also require enough number of sequencing read counts (m6A positive + m6A negative >= 50) to reliably calculate m6A levels as we previously described (Nature Methods. 2016,13(8):692-8). Therefore, there are only 223 pseudogenes with reliable m6A levels in GM12878 cells. We have briefly explained this issue in the RESULTS (page 7).

2.If I understand correctly, authors improve their mapping of the reads to study the m6A levels compared to previous study. They consider the new approach more reliable. Is there a way to confirm that this is the case?

Author Response: Thanks for the reviewer’s comment! Our mapping procedure considered SNPs in the mapping. Because GM12878 is a deeply sequenced individual of 1000 genome project, there we can require no mismatch except known SNPs. As shown in the Additional file 1: Fig. S1d and e, there are two examples that conventional mapping misaligned the reads between pseudogenes and cognate coding genes due to because it recognizes the SNPs as mismatches, however, our mapping procedure can correctly map these reads.

3.In figure 1b, if seems that what is different are cognate coding genes of processed pseudogenes. These cognate coding genes are the ones that show low m6A level percent. Are these cognate coding genes different in some way that explains their low m6A level?

Author Response: Thanks for the reviewer’s comment! Processed pseudogenes were generated by retrotransposition. As previously reported, the retrotransposed mRNAs tend to be (1) stable in cytoplasm; (2) resistant to NMD; (3) translated on free cytoplasmic ribosomes (Trends Genetics.2006,22(2):69-73). Therefore, the cognate coding genes of processed pseudogenes are usually very stable RNAs that less likely to be modified by m6A, which promotes RNA decay. We have added the following sentence in BACKGROUND (page 4):

“*As previously reported, the retrotransposed mRNAs tend to be stable transcripts translated on free cytoplasmic ribosomes*”

4.In figure 2 g, it seems that processed pseudogenes evolve high methylation level very quickly. Is this the case?

Author Response: Thanks for the reviewer’s comment! Yes, the processed pseudogenes evolve high methylation level very quickly. Considering that the peak intensities of m6A peaks are overall conserved between human and mouse (Cell Stem Cell.2014,15(6):707-19), our data do support processed pseudogenes evolve high methylation level very quickly. We have added the following sentence in the RESULTS (page 9):

“*Considering that the peak intensities of m6A peaks are overall conserved between human and mouse, the above results support that pseudogenes especially processed pseudogenes were methylated lowly at birth and evolved high methylation levels quickly*.”

5.In the past, even processed copies of genes that do not have disablements have been called processed pseudogenes. So, authors should clarify what kinds of disablements the pseudogenes considered in this work have.

Author Response: Thanks for the reviewer’s comment! The pseudogenes we used are actually all in GENCODE pseudogene annotation, which requires at least one of the following characteristics: “ (1) a premature stop codon relative to parent CDS - can be introduced by nonsense or frame-shift mutation; (2) a frame-shift in a functional domain - even where the length of the resulting CDS is similar to that of the parent CDS; (3) a truncation of the 5' or 3' end of the CDS relative to the parent CDS; (4) a deletion of an internal portion of the CDS relative to the parent CDS. Processed pseudogene loci lacking disabling mutations are annotated as 'pseudogene' when they lack locusspecific transcriptional evidence.” (Genome Biology,2012,13:R51). We have clarified the disablements in the METHODS (page 19).

Other comments:

1.Line 77-80. Processed and unprocessed pseudogenes are duplicates. Processed pseudogenes are duplicates that were produced through an mRNA intermediate and unprocessed pseudogenes are duplicates that were produced through an DNA intermediate.

2.Line 82. I would say "…lack of intronS."

3.Line 144. Remove et al.

4.Line 174. I would say "The m6A motifs on pseudogenes EVOLVED under positive natural selection."

5.Line 314. I think authors mean "neutral"

Author Response: Thanks for the reviewer’s comment! We have fixed all these issues.

**2^nd^ round**

**Reviewer 1**

Most of reviewer's concerns were satisfactorily addressed in the revised manuscript. I have only one minor comment.

1. Throughout manuscript, "Nonsense Mediated Decay" should be changed to "Nonsense-mediated mRNA decay".

**Reviewer 2**

No further comments.

**Reviewer 3**

Authors have addressed the questions I had.
